# A versatile, bioengineered skin reconstruction device designed for use in austere environments

**DOI:** 10.3389/fbioe.2023.1208322

**Published:** 2023-06-08

**Authors:** Joachim G. S. Veit, Morgan Weidow, Monica A. Serban

**Affiliations:** ^1^ Serban Lab, Department of Biomedical and Pharmaceutical Sciences, University of Montana, Missoula, MT, United States; ^2^ Montana Biotechnology Center (BIOTECH), University of Montana, Missoula, MT, United States

**Keywords:** austere environment, skin reconstruction device, wound healing, biomaterial, microneedles, silk fibroin, hyaluronic acid

## Abstract

Austere environments in which access to medical facilities, medical personnel, or even water and electricity is limited or unavailable pose unique challenges for medical device product design. Currently existing skin substitutes are severely inadequate for the treatment of severe burns, chronic wounds, battlefield injuries, or work-related injuries in resource-limited settings. For such settings, an ideal device should be biocompatible, bioresorbable, promote tissue healing, not require trained medical personnel for deployment and use, and should enable topical drug delivery. As proof of concept for such a device, silk fibroin and an antioxidant hyaluronic acid derivative were chosen as primary constituents. The final formulation was selected to optimize tensile strength while retaining mechanical compliance and protection from reactive oxygen species (ROS). The ultimate tensile strength of the device was 438.0 KPa. Viability of dermal fibroblasts challenged with ROS-generating menadione decreased to 49.7% of control, which was rescued by pre-treatment with the hyaluronic acid derivative to 85.0% of control. The final device formulation was also tested in a standardized, validated, *in vitro* skin irritation test which revealed no tissue damage or statistical difference from control. Improved topical drug delivery was achieved via an integrated silk fibroin microneedle array and selective device processing to generate crosslinked/through pores. The final device including these features showed a 223% increase in small molecule epidermal permeation relative to the control. Scaffold porosity and microneedle integrity before and after application were confirmed by electron microscopy. Next, the device was designed to be self-adherent to enable deployment without the need of traditional fixation methods. Device tissue adhesive strength (12.0 MPa) was evaluated and shown to be comparable to a commercial adhesive surgical drape (12.9 MPa) and superior to an over-the-counter liquid bandage (4.1 MPa). Finally, the device’s wound healing potential was assessed in an *in vitro* full-thickness skin wound model which showed promising device integration into the tissue and cellular migration into and above the device. Overall, these results suggest that this prototype, specifically designed for use in austere environments, is mechanically robust, is cytocompatible, protects from ROS damage, is self-adherent without traditional fixation methods, and promotes tissue repair.

## 1 Introduction

Skin functions as our primary barrier to the environment and provides protection from pathogens, allergens, radiation, and mechanical injury. It also plays an important role in metabolism, fluid regulation, and thermal homeostasis. Disruption of the skin’s integrity can be life-threatening and as such, repairing damage from insults such as burns, trauma, medical procedures, and others, is critical. Civilian modern medical facilities or military tertiary care facilities are well equipped to successfully handle such procedures. However, wound repair and skin reconstruction in resource-limited settings poses unique challenges and drastically changes design requirements for adequate solutions. As an example, in combat areas, repair and reconstructive care is typically delayed until transfer to adequately equipped tertiary care centers rather than being addressed in primary or secondary care centers. This delayed care can lead to severe wound deterioration and is associated with increased morbidity and mortality (Bhandari et al., 2012; Driscoll et al., 2018). Within a civilian context, rural areas and communities of lower socioeconomic status report a higher incidence and severity of burn injuries than wealthy or urban areas, which is exacerbated by a lack of specialized care centers and trained medical personnel (Vidal-Trecan et al., 2000; Chamania, 2010). The World Health Organization reports that 180,000 deaths occur every year due to burns, primarily in low- and middle-income countries, and burns are a leading cause of disability-adjusted life-years in these areas (World Health Organization, 2018). Regarding chronic wounds, a similar disparity is observed in these underserved populations in which the risk of major amputation associated with chronic wounds is 50% higher in rural patients when compared to their urban counterparts, which is further accentuated in low-income countries by ulcerative skin diseases and neglected tropical diseases (Sutherland et al., 2020; Sen, 2021; Toppino et al., 2022). These facts clearly underline the need for products and wound care mitigation strategies specific to austere, resource-limited settings, while producing optimal therapeutic outcomes.

Although there are numerous wound healing products on the market today, none are suitable as critical care products for use in resource-limited settings (Jeschke et al., 2018; Snyder et al., 2020). Allografts, autografts, synthetic, and semi-synthetic devices are commercially available, but all require application and use in a properly equipped medical facility by highly trained medical personnel. Additionally, many of them require multiple surgical steps which are unfeasible in resource-limited settings.

We sought to develop a skin reconstruction device which acts as a protective barrier upon application while promoting concurrent tissue healing and device biodegradation to allow autologous remodeling of wounded tissue. Designed specifically for resource-limited settings, the device aims to simplify deployment, increase user-friendliness by removing requirements for sutures or other traditional fixation methods, allow patient-specific topical drug application, facilitate biointegration into healing tissue to reduce the need for sequential surgical procedures, and allow for use by any first responder or care provider.

For the device formulation, we chose well-characterized biodegradable natural polymers with good environmental stability, specifically silk fibroin (SF) and hyaluronic acid (HA) for optimal tissue integration, therapeutic outcome, and cold-chain independence (Holland et al., 2019). We also used another well-characterized material, polyvinyl alcohol (PVA), as a transient structural stabilizer and porogen. To ensure accelerated tissue healing, we tailored the properties of HA via chemical conjugation with D-methionine (Dmet) to yield an HA-methionine conjugate (HAM) capable of mitigating oxidative stress, which often is associated with inflammation and delayed wound healing (Deng et al., 2021; Wang et al., 2023). To enable topical drug delivery, the device scaffold was processed to be macro- and micro-porous, and was equipped with an integrated microneedle array for profound tissue access. This prototype was intentionally designed drug-free to allow for user and patient-specific drug selection, which translates to increased versatility within austere environments by avoiding the need to possess multiple formulations of the device. Moreover, from a product perspective, the drug-free design would be regulated by the US Food and Drug Administration as a biomedical device rather than a combination product (Serban, 2016). Finally, to maximize user-friendliness and ease of deployment, the device was rendered self-adherent upon contact with the wound and can be easily set in place without the need for traditional fixation systems.

To optimize this concept’s utility for resource-limited settings, the formulation and assembly processes were intended to be simple, efficient, and effective, thus ensuring downstream cost-efficiency, easy scalability and, ideally, cold-chain independence, although this was not evaluated in the current study. The data presented here serves as proof of concept for a unique skin reconstruction device purposely designed to address the unique unmet needs of critical wound care and skin reconstruction in settings where currently existing products and technologies are irrelevant.

## 2 Materials and methods

### 2.1 Chemicals and antibodies

The following chemicals were used in this study: polydimethylsiloxane (PDMS) (Dow Silicones Corporation, Sylgard 184); polyethylene glycol (PEG) 10 kDa (Sigma-Aldrich, 81280); Anhydrous Sodium Carbonate (EMD, SX0395-1); polyvinyl alcohol (PVA) 146–186 kDa (Acros Organics, 183160010); anhydrous lithium bromide (Acros Organics, 453980010); Dulbecco’s phosphate buffered saline (Corning, 21-031-CV); fluorescein disodium (Alfa Aesar, J61549.22); D-methionine (Alfa Aesar, B21213); hyaluronic acid (Lifecore, HA1M-5); iodoacetic acid (Acros Organics, 122280250); BupH MES buffered saline (Thermo, 28390); 1-Ethyl-3-(3-dimethylaminopropyl) carbodiimide (EDC) (Thermo, 22980); (3-4,5-dimethyl thiazole 2-yl) 2,5-diphenyltetrazoliumbromide (MTT); Mowiol 4-88 (EMD Millipore, 475904). glycerol (Fisherbrand, G33-500); 1,4-diazobicyclo-[2.2.2]-octane (Alfa Aesar, A14003); O-phthalaldehyde (OPA) (TCI, P0280); mercaptoacetic acid (Fisherbrand, AC125430010); 1-heptanesulfonate monohydrate (Fisherbrand, AA1521418).

The following antibodies were used in this study: mouse anti-human keratin 14 (Abcam, ab7800, RRID:AB_306091) was diluted 1:1000; rabbit anti-human vimentin (Abcam, ab92547, RRID:AB_10562134) was diluted 1:250; both secondary antibodies, donkey anti-rabbit Alexa Fluor Plus 555 (Invitrogen, A32794, RRID:AB_2762834) and goat anti-mouse Alexa Fluor 647 (Abcam, ab150115, RRID:AB_2687948), were diluted 1:1000; DAPI (Thermo Scientific, 62248) was diluted 1:1000.

### 2.2 Silk isolation

Silk fibroin (SF) was extracted from *Bombyx mori* silk yarn (Bratac, Brazil) as previously described (Rockwood et al., 2011; Johnston et al., 2018). Briefly, yarn was boiled for 30 min in sodium carbonate (0.02 M) to remove sericin, then rinsed and dried. Extracted SF was then dissolved in lithium bromide (9.3 M) at 20% w/v for 4 h at 60°C. This was then transferred to a Slide-A-Lyzer G2 3.5k MWCO dialysis cassette (Thermo Scientific, 87725) and dialyzed against deionized water (3–4 L) for 48 h (8-10 water changes) to yield 6%–8% (w/v) SF. To further concentrate, the cassette was then transferred to a solution of 10 kDa PEG (20% w/v) in deionized water until the desired concentration (14%–15% SF) was reached. Any precipitated solids were removed from the solution by centrifugation at 4°C, 3600 RCF for 2 × 20 min and decanting the solution. Concentration was determined by adding SF solution (1.0 mL) into a MJ33 moisture analyzer (Mettler Toledo) and recording the mass of the solid remaining after drying. Concentrated SF solutions were stored at 4°C until use.

### 2.3 HAM synthesis

Carboxy methyl hyaluronic acid (CMHA) was synthesized as previously described (Arrigali and Serban, 2022) from HA, which increases available carboxy moieties for subsequent d-methionine (Dmet) conjugation. Briefly, HA (0.4 g) was ground into a fine powder in a glass mortar and pestle, then dissolved in sodium hydroxide (4 mL, 45% w/v) with stirring for 2 h at room temperature (RT). Isopropanol (30 mL) was then added followed by iodoacetic acid (0.423 M) in isopropanol (10 mL) and stirred for 2 h at RT. Isopropanol was removed by vacuum filtration. Then, the resulting solid was dissolved in deionized (DI) water (40 mL) and the pH was neutralized (∼7.0) before the solution was dialyzed against DI water for 72 h (12-15 water changes) with a Slide-A-Lyzer 3.5k MWCO dialysis, cassette (Thermo Scientific, 66130). After dialysis, the solution was frozen, lyophilized, and stored at −20°C in an airtight container until use.

Conjugation of Dmet to CMHA (HAM) was done as previously described (Arrigali and Serban, 2022). Briefly, CMHA (50 mg) was dissolved in MES buffer (10 mL). Dmet (220 mg) was then added, followed by EDC (100 mg). This mixture was allowed to react overnight with stirring at RT, then was neutralized with NaOH (1 M) and dialyzed against DI water for 72 h. After dialysis, the HAM solution was frozen, lyophilized, and stored at −20°C in an airtight container until use.

Modification of CMHA and HAM was confirmed by ^1^H-NMR on a Bruker 400 MHz using product in deuterated water (10 mg mL^−1^). Carboxymethylation efficiency was determined using quantitative ^1^H-NMR to normalize the three carboxymethyl methylene protons (4.1 ppm) to the two methyl protons of the N-acetylglucosamine of HA (1.95 ppm) (Supplementary Figure S1).

### 2.4 Scaffold casting

Varying concentrations of SF and HAM were combined in conical tubes by gently pipetting up and down followed by brief immersion in a sonicating water bath to homogenize. A 32 × 32 × 1 mm (or 32 × 32 × 0.5 mm) deep well was cast in PDMS from a simple template made in Fusion 360 software and printed on a Form 3 (Formlabs) SLA 3D printer using Clear V4 photopolymer ink. These PDMS molds were pre-chilled, then used to cast 1 mm (or 0.5 mm for wound healing) thick SF/HAM scaffolds. The scaffolds were frozen in a −80°C freezer overnight, then lyophilized for 24 h. Following lyophilization, the SF was made water insoluble by inducing physical crosslinking via β-sheet formation by soaking in 90% ethanol for 1 h (scaffolds used for tension testing were soaked overnight to match ethanol treatment of final device). β-sheet formation (Chen et al., 2009; Love et al., 2019) indicated by the amide I peak at 1620 cm^-1^ was confirmed by FTIR using a Nicolet iS50 FTIR with ATR (Supplementary Figure S2).

### 2.5 Microneedle array

A microneedle (MN) array template was designed using Fusion 360 software (Autodesk). The 21 × 36 MN array consisted of 400 µm tall conical MNs with a base diameter of 120 µm and pitch of 600 µm. The needle profile consists of 3 facets: the first 110 µm from the tip has walls angled at 15° from vertical to allow for a sharp, low-penetration force tip; this transitions to a main shaft with more gradually sloped sides, 6° from vertical, to provide increased compressive support and lateral rigidity; finally, a 25 µm radius fillet was placed at the intersection of the MN shaft and the base to prevent stress concentrations. This design was sent to the University of Utah Nanofabrication Lab (Salt Lake City, UT) for two-photon polymerization (2PP) 3D printing on a Photonic Pro GT2 (Nanoscribe) using IP-S resin and a ×10 objective. Following printing, a negative of the MN array was cast using Sylgard 184 polydimethylsiloxane (PDMS) as per manufacturer’s recommendation and allowed to fully cure overnight at 35°C before separating from the MN template.

PVA was dissolved at 8% (w/v) in nanopure water and stirred at 90°C until solubilized. Concentrated SF was diluted to 8% and combined with PVA at a 7:3 (SF:PVA) ratio by volume, pipetted to mix, then submerged 15–20 s in sonicating water bath to emulsify and degas the mixture. This SF:PVA solution was then cast into the PDMS MN negative mold and centrifuged at 3600 RCF for 5 min to completely fill the MN negative mold and remove any trapped air. This was then allowed to air dry overnight to form a film.

Characterization of MN and MN equipped devices was performed by scanning electron microscopy (SEM) on a Thermo Scientific Phenom ProX SEM with SED/EDS. Some SEM samples were coated in 5 nm gold by plasma deposition in air using a LUXOR Goldcoater to improve image resolution. Uncoated samples were also imaged to confirm that no changes were induced by gold coating.

### 2.6 Device assembly

SF scaffolds were lightly brushed with a solution of SF (6%–8% w/v) on one side, placed on the backside of the MN film still within the PDMS mold, then pressed with a glass coverslip (to provide even pressure) and a 30 g weight while it dries. After drying, the entire assembly is submerged in 90% ethanol overnight to induce β-sheet formation. The device is then allowed to air dry completely. A final thin layer of tissue adhesive aerosolized SF was applied to the surface of the MN using a TLC reagent sprayer (Kimble, 422530-0025) loaded with a SF solution (6%–8% w/v), then allowed to air dry. This random coil-abundant, water soluble layer of SF reactivates when exposed to moisture and acts as an adhesive, as has been previously demonstrated (Johnston et al., 2018), to secure the device.

### 2.7 Tensile strength testing

Scaffolds or assembled devices were cut into 10 × 30 × 1 mm dog bone-shaped sheets for tension testing using a hobby knife and custom 3D-printed stencil. Samples were soaked for 30 min in PBS, lightly pressed between two lab tissues to remove all excess liquid, then the sample was clamped into the tension fixture (Supplementary Figure S3A) of a Discovery HR-2 hybrid rheometer (TA Instruments, New Castle, DE). Tensile force was evaluated at room temperature with a constant linear rate of 166.67 μm s^−1^ until failure. Any samples which failed at the fixture interface were considered invalid and removed from the data. Ultimate tensile strength (UTS) testing was performed on eight distinct sample replicates.

### 2.8 Reactive oxygen species protection assay

Primary neonatal fibroblasts were purchased from ATCC (PCS-201-010) and cultured in fibroblast basal medium supplemented with low-serum fibroblast growth kit and 0.5% penicillin-streptomycin-amphotericin B (ATCC: PCS-201-030, PCS-201-041, PCS-999-002, respectively) using manufacturer’s protocols. After trypsinization and counting using a Countess automated cell counter (Thermo Scientific), fibroblasts were seeded at 6.0 × 10^3^ cells per well into a 96-well plate, incubated in a 37°C, 5% CO2 humidified incubator for 24 h before beginning treating with appropriate treatment for 24 h. After 24 h, the treatment was replaced with menadione (10 µM), or vehicle (0.1% ethanol in media) in control group, and returned to incubator for 5 h. Wells were rinsed with growth media then the Cell-titer 96 Aqueous One Solution Cell Proliferation Assay kit (Promega, G3580) was run using manufacturer’s protocol and absorbance at 490 nm was read on a Cytation 5 microplate reader (Biotek). Reactive oxygen species (ROS) protection assay was performed in biological quadruplicates measured in technical duplicates.

### 2.9 Skin irritation testing

Skin irritation testing (SIT) was performed following OECD TG439 protocols and using *in vitro* EpiDerm tissues (Mattek Corporation, EPI-200-SIT). Briefly, complete devices were frozen in liquid nitrogen, then ground into a fine powder using a glass mortar and pestle. A drop of DPBS (25 µL) was placed on the epidermal surface of each test tissue, followed by 25 mg of the powdered device. Tissues were exposed to a negative control (DPBS), positive control (5% SDS solution), or the test substance for 60 ± 1 min, thoroughly washed, then further incubated for 42 h. Cell viability was then determined by reduction of MTT using absorbance at 570 nm on a Cytation5 microplate reader. SIT was performed as directed by the standardized protocol in biological triplicates measured in technical duplicates.

### 2.10 Fibroblast migration

Scaffolds (1 mm thickness) were prepared from a solution of SF (12% w/v) with HAM (0.2% w/v), frozen and lyophilized as described above, then rendered insoluble in 90% ethanol. Disks (8 mm diameter) were punched from the scaffolds, then transferred to sterile 24-well plates in a laminar flow cell culture hood where they were allowed to completely dry in sterile conditions for 3 days. The scaffolds were pre-soaked in fibroblast growth media for 1 h and placed in cell culture inserts (Millipore PIHP01250) to keep the scaffold surface above the surface of the media. A 5 µL drop containing 4.0 × 10^5^ fibroblasts was gently pipetted onto the center of the scaffold. These were placed in a 37°C, 5% CO_2_ humidified incubator overnight to allow cells to properly adhere, then additional media was added to each well to fully submerge the scaffolds. Growth media was subsequently changed every 2 days. Scaffolds were collected on day 2 and day 7 after initial seeding, fixed, then immuno-labeled for confocal imaging (see below). Fibroblast migration was performed on four distinct samples for each collection day.

### 2.11 Adhesive testing

Complete devices were prepared as indicated before. Circular 8 mm diameter patches of the devices or Steri-Drape^TM^ (3M Healthcare, St. Paul, MN) were cut out with a biopsy punch (Accuderm, Fort Lauderdale, FL). A small square of natural sheep chamois leather (Amazon, Seattle, WA) was cut out, moistened, then all extra water was squeezed out with a lab tissue. The samples were then placed onto the leather with the adhesive against the leather and a ∼30 g weight was placed onto the device to provide downward pressure as it dried overnight. The following day, a small drop of cyanoacrylate glue (Gorilla Glue Company, Cincinnati, OH) was placed onto a custom 3D printed adapter (Supplementary Figure S3B) and stuck to the top of the adhered sample. For the New-Skin^TM^ liquid bandage (Advantice Health, Cedar Knolls, NJ) samples, a thin coating of liquid bandage was applied directly to the 3D printed adapter, which was then pressed onto a square of chamois leather and allowed to dry for the manufacturer-recommended 5 min. The samples were clamped into the top of the tension fixture of the DHR-2, then a small drop of glue was placed onto a similar 3D printed adapter clamped to the bottom of the tension fixture. The fixture was automatically lowered until the glue contacted the chamois leather. This was then held at a constant force of 0.5 N for 15 min to allow the glue to dry. After 15 min, the tension fixtures began to separate at a rate of 100 μm s^-1^ until the device had completely separated from the leather. All samples were only considered valid if the separation occurred at the interface of the adhesive and the leather (this was true for all device samples tested; none failed at any other location). The absolute value of the peak tension (N) experienced before failure was divided by the adhesive surface area of the device (m^2^) to calculate the tensile strength (in Pa) of the adhesive. Adhesive testing was performed on six distinct sample replicates.

### 2.12 Drug permeation *in vitro* reconstructed human epidermis

Reconstructed human epidermis (RHE) were grown *in vitro* and cultured at air-liquid interface for 11 days as previously described in detail (Veit et al., 2021a; Veit et al., 2021b) using primary neonatal epidermal keratinocytes (Gibco, C-001–5C). The fully developed RHE were transferred to a 12-well plate containing 600 µL DPBS per well. A 4 mm diameter device was placed into the center of the RHE and gently pressed down with a cotton tipped swab to puncture the skin with the MN. The MN-free scaffold group was also pressed with a cotton swab with equal force to control for any non-MN associated damage affecting drug permeation. Fluorescein disodium (1 mM) in DPBS (10 µL) was applied onto the top of each scaffold and the tissues were incubated between timepoints in a dark, humidified, 37°C incubator. At each timepoint, the RHE were transferred to a new well containing fresh DPBS. The previous timepoint was collected in a microcentrifuge tube and stored at −20°C until analysis. When necessary, samples were diluted to within the linear range of the standard curve and the total fluorescein permeated was determined by fluorescence on a Cytation5 (Biotek) microplate reader (ex. 490 nm; em. 515 nm). Drug permeation assay was performed in biological quadruplicates measured in technical duplicates.

### 2.13 Full-thickness in vitro skin wound healing assay

Complete devices were punched into 3 mm diameter disks during the 90% EtOH soaking step of production, then transferred into a covered, sterile TC plate in a flowing tissue culture hood to dry overnight.

EpiDerm full-thickness (EFT) *in vitro* skin models were purchased from Mattek Corporation (EFT-412). Following the manufacturer’s recommended overnight equilibration period in a 37°C, 5% CO_2_ humidified incubator, tissues were wounded using a 3 mm biopsy punch (Accuderm, Fort Lauderdale, FL) and the resulting tissue plug was removed with fine-tipped sterile tweezers. The prepared sterile 3 mm devices were briefly soaked in DPBS, then gently placed into the induced EFT wounds.

These tissues were allowed to grow for 20 days, with media changes every 1–2 days using the supplied manufacturer’s media. The tissues were then fixed and processed for histological analysis (see below).

### 2.14 Histology and immuno-labeling of tissues

RHE and EFT tissues were fixed overnight (4% formaldehyde with 1% acetic acid in DPBS), transferred to histology cassettes, then processed for paraffin embedding in an ASP300S tissue processor (Leica Biosystems, Deer Park, IL). Paraffin embedded tissues were cut down on a microtome until the device was just exposed, then an additional 1.0 mm was removed to allow for a series of 6–10 µm sections to be made in the approximate center of the applied device. The slide-mounted sections were then either stained with hematoxylin and eosin (H&E) or deparaffinized and rehydrated for immuno-labeling using an Autostainer XL (Leica) robot. All tissue processing and sectioning equipment was provided by the Center for Environmental Health Sciences Molecular Histology and Fluorescent Imaging Core at the University of Montana.

Antigen retrieval of deparaffinized sections was done by placing slides in pH 6.0 citric acid (2.2 g L^-1^) within a double boiler for 20 min. Slides were rinsed in PBS, then blocked for 1 h in a buffer containing glycine (0.1 M), bovine serum albumin (BSA) (1% w/v), and triton X-100 (0.02% v/v), in PBS. Primary antibodies were diluted in BSA (0.2%) and triton X-100 (0.02%) in PBS (PBT buffer) and applied to the slides for overnight incubation at 4°C in a humidity chamber. The following day, slides were rinsed 3 × 3 min in PBT buffer with gentle orbital shaking. Secondary antibodies diluted in PBT buffer were incubated for 1 h at room temperature in a humidity chamber. Slides were again rinsed 3 × 3 min in PBT buffer, then incubated for 10 min with DAPI in PBT buffer before a final 3 × 5 min rise in PBS. Coverslips were mounted with Mowiol + DABCO mounting media (10% w/v Mowiol 4-88, 25% w/v glycerol, 0.1 M Tris (pH 8.5), and 2.5% w/v 1,4-diazobicyclo-[2.2.2]-octane).

H&E stained tissues were imaged on a DMI3000B (Leica Microsystems, Deerfield, IL) inverted widefield microscope with a Leica DFC450C camera. Immuno-labeled samples were imaged on a Leica Stellaris 5 confocal microscope.

### 2.15 Statistical analysis

Statistical analysis was performed as described in respective figure captions using GraphPad Prism software (v9.4.1). Sample sizes in figure captions refer to the number of distinct sample replicates and do not include technical replicates. Technical replicates were combined prior to statistical analysis.

## 3 Results

### 3.1 Device design and production

The complete device formulation consists of SF as the main structural component, HAM as the main bioactive component, and PVA for transient structural stability and porosity. Physical and biological properties of HAM can be found in Supplementary Materials (Supplementary Figures S4, S5).

**FIGURE 1:**
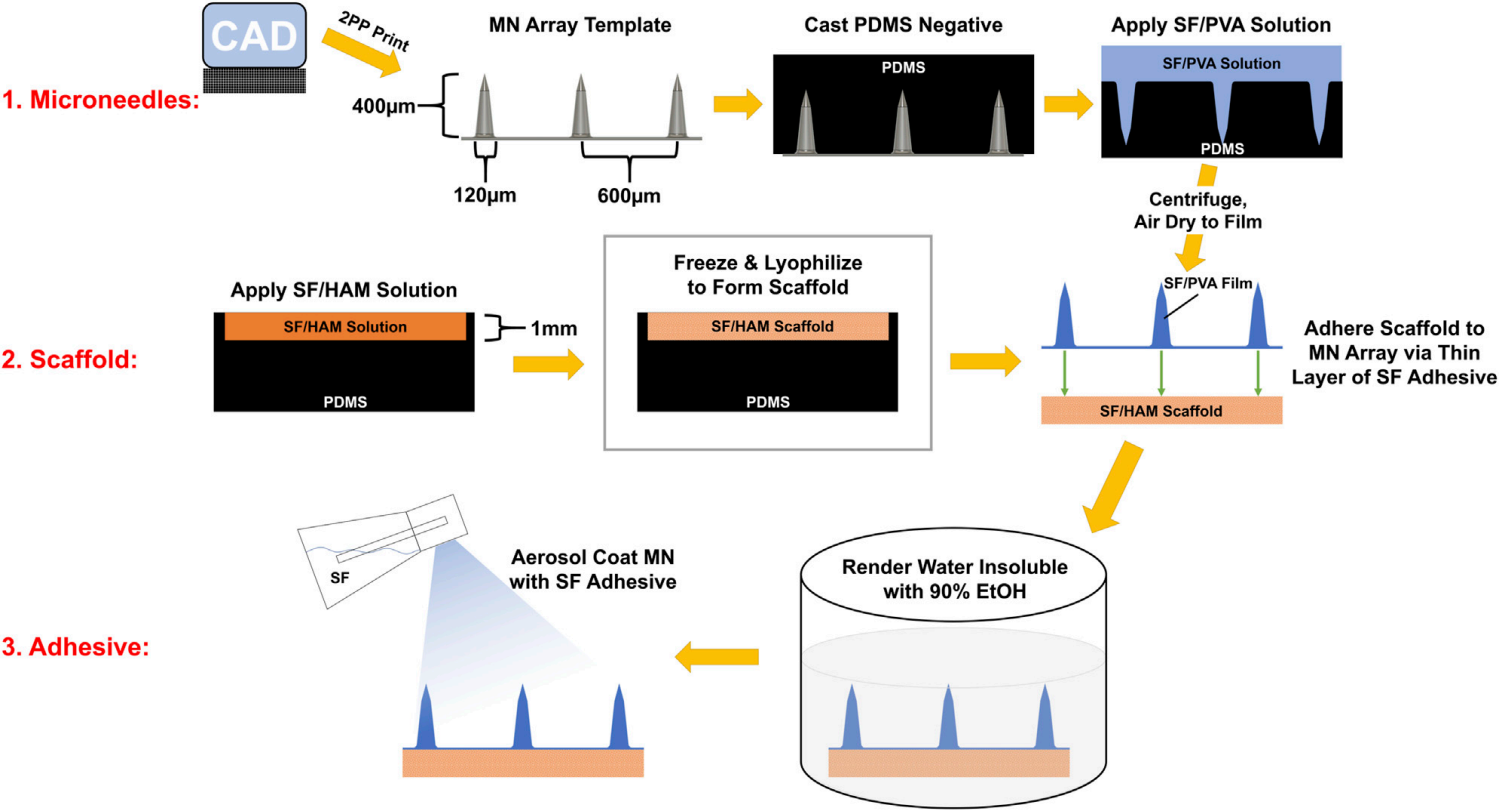
Infographic of the device assembly procedure. MN array template was manufactured with photopolymer resin on a 2PP 3D printer. A PDMS negative mold of this template was cast and used cast SF/PVA MN arrays. Separately, a SF/HAM solution is frozen and lyophilized to form a 1 mm thick scaffold. The scaffold and MN array are bonded together, then this assembly is rendered water insoluble by inducing β-sheets in SF. Finally, SF adhesive is aerosol coated onto the surface of the MNs.

**FIGURE 2:**
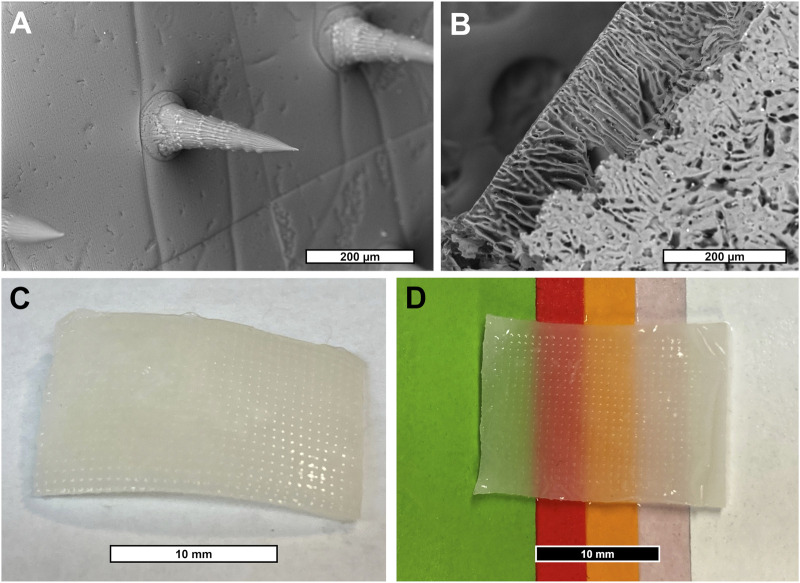
**(A)** SEM micrograph of SF/PVA MN array. **(B)** SEM micrograph of SF/HAM scaffold surface and partial cross section. **(C)** Photograph of hydrated device showing MN surface and **(D)** demonstrating the devices’ translucent property allowing it to inherit the underlying colors.

As a first step in creating a complete device, a negative mold of conical MNs was designed and manufactured as described in Section 2.5 from a micron-scale 3D printed template (Figure 1). Next, a mixture of SF and PVA was cast into a negative MN mold and allowed to dry into a film of sharp MNs with a tip radius of ∼2 µm (Figure 2A, Supplementary Figure S6). Separately, a porous scaffold (Figure 2B) was generated by freezing and lyophilizing a solution of SF and HAM placed in a 1 mm deep cuboidal negative mold. This scaffold was subsequently adhered to the dried MN array with SF adhesive, rendered water-insoluble by ethanol-induced β-sheet formation (confirmed by FTIR, Supplementary Figure S2), then coated with a thin layer of aerosolized SF tissue adhesive. This three-component complete device (scaffold, bonded MN array, and aerosolized SF adhesive) is stored dry until ready for use when it can be quickly activated by rehydration. For the purposes of this study, “complete device” (Figure 2C), refers to the final selected formulation of the device which consists of a scaffold made from a solution of 12% (w/v) SF and 0.2% (w/v) HAM which is adhered to a MN array containing a 7:3 mass ratio of SF to PVA, which has been rendered water insoluble then coated in SF adhesive. Interestingly, once hydrated, the complete device appears translucent and capable of adopting any underlying colors (Figure 2D).

### 3.2 Device characterization

#### 3.2.1 Mechanical properties

**FIGURE 3:**
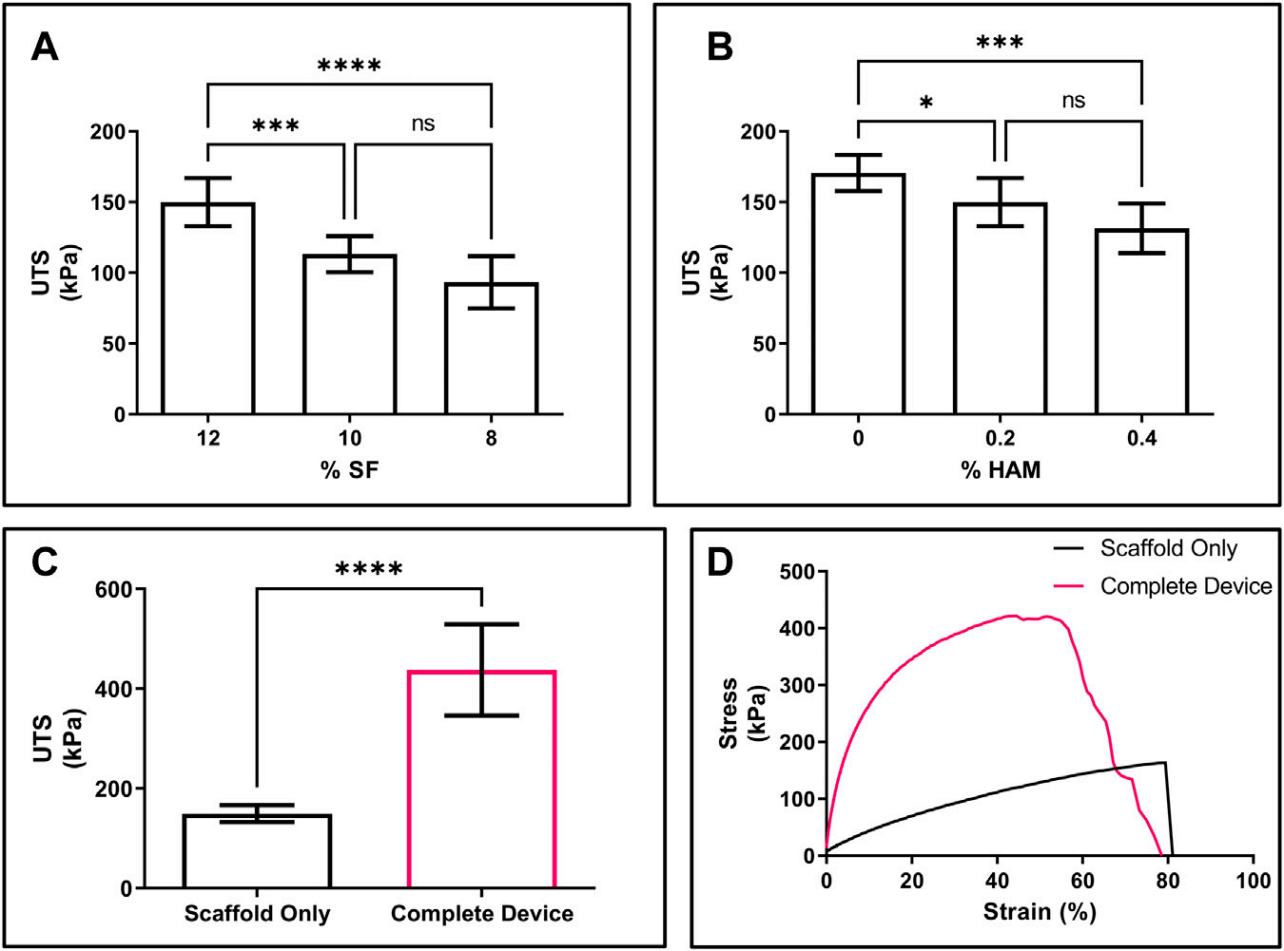
Mechanical properties of scaffold and device. **(A)** UTS of scaffold made from solutions containing varying concentrations of SF with 0.2% HAM. **(B)** UTS of scaffold from solutions containing 12% SF and varying concentrations of HAM. **(C)** UTS of the final formations of the scaffold only (black) compared to the complete device (pink). **(D)** Representative stress-strain curve for scaffold only (black) compared to the complete device (pink). **(A,B)** n = 8; one-way ANOVA with Dunnett’s correction for multiple comparisons; *p < 0.05, ***p < 0.001, ****p < 0.0001. **(C)** n = 8; 2-sided *t*-test; ****p < 0.0001. *NS, not significant.*

To determine the best balance between tensile strength, porosity, and HAM content, while retaining a compliant, skin-like feel (Supplementary Video S1), scaffolds were made with solutions containing varying concentrations of SF and HAM. For the scaffolds, both a decrease in SF (Figure 3A) and an increase in HAM (Figure 3B) content resulted in decreased UTS. The elongation at break for the final scaffold formation (12% SF + 0.2% HAM) was 78.3% ± 10.3% (mean ± SD), and there were no statistically significant differences in elongation at break from varying SF or HAM content in the scaffold (Supplementary Figures S7A, B). The complete device showed a significant increase in UTS (438.0 ± 91.5 KPa; mean ± SD) compared to the scaffold alone (149.9 ± 17.0 KPa) (Figure 3C) and displayed a steeper rise in the stress-strain curve, indicating a larger elastic modulus (Figure 3D) due to the additional components (MN array film and SF adhesive). Compared to the scaffold only, the elongation at break of the complete device was 42.9% ± 12.5% (Supplementary Figure S7C), likely due to the lower elasticity of the MN film.

#### 3.2.2 ROS protection, cytocompatibility, and skin irritation testing

**FIGURE 4:**
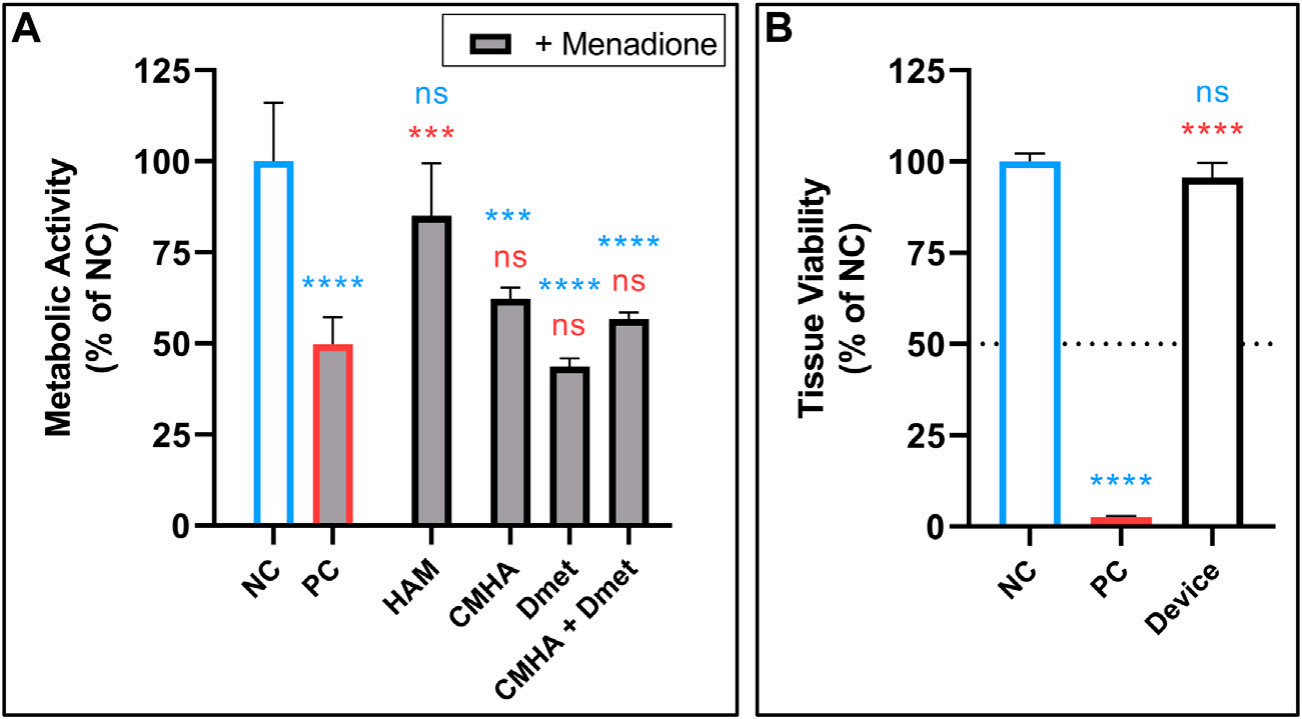
**(A)** ROS protection of fibroblasts by treatment with 2 mg mL-1 HAM or equivalent concentrations of CMHA alone, Dmet alone, or a blend of CMHA + Dmet. Followed by treatment with ROS-generating menadione (indicated by grey fill). n = 4. **(B)** Skin irritation test of NC (vehicle), PC (5% SDS), and final device formulation (25 mg, powdered) performed in 3D *in vitro* human epidermis. Dotted line shows cutoff threshold indicating whether test compound is considered a possible irritant. n = 3. **(A,B)** Mean +SD; one-way ANOVA with Tukey’s correction for multiple comparisons vs. NC (blue) or PC (red); **p < 0.01, ***p < 0.001, ****p < 0.0001. *NC, negative control; NS, not significant; PC, positive control; SDS, sodium dodecyl sulfate.*

The ability of HAM to protect against ROS was evaluated using an MTS colorimetric assay, which measures metabolic activity and is reflective of cell viability. Primary human fibroblasts were pretreated with the indicated material or a vehicle control, then challenged by treatment with ROS-generating menadione. The menadione-challenged positive control (PC) significantly reduced fibroblast activity relative to the negative control (NC). This reduced activity was reversed and no longer significantly different than NC in the cells pre-treated with either 2 or 1 mg mL-1 HAM (Figure 4A, Supplementary Figures S5B). CMHA-alone, Dmet-alone, and a non-conjugated blend of the two, all in HAM-equivalent concentrations (based on mass ratio of CMHA to Dmet in HAM), were found to have no significant protective effect, highlighting the importance of chemical conjugation between HA and Dmet.

Next, SIT was performed according to validated protocols (OECD TG439) using 25 mg of the complete device in powdered form. No evidence of skin irritation was found, nor was any statistically significant change in cell viability detected relative to the untreated control (Figure 4B).

**FIGURE 5:**
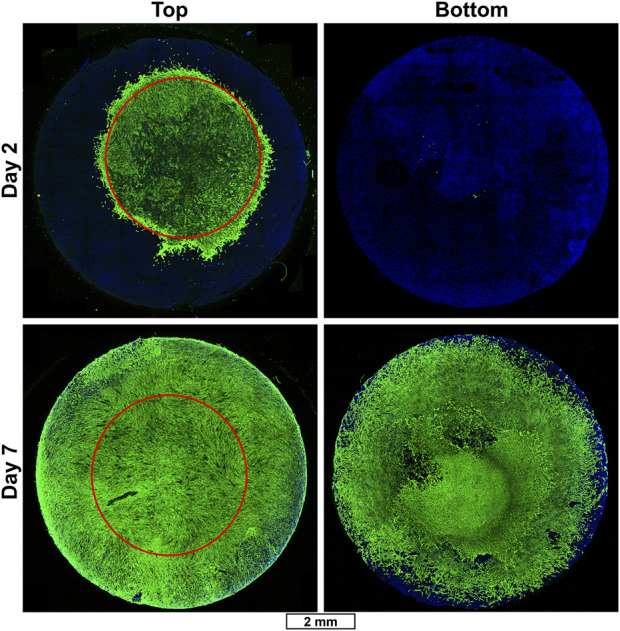
Fibroblast migration in SF/HAM scaffold. Representative immunofluorescent confocal micrographs of SF/HAM scaffolds cultured for 2 (top row) or 7 (bottom row) days following initial seeding with a 5 µL drop of primary fibroblasts directly onto the top center surface of the scaffold. Red circle shows approximate initial seeding area to illustrate extent of migration. Scale bar applies to all images. DAPI and auto-fluorescent SF (blue); vimentin staining identifies fibroblasts (green).

In addition, the device’s ability to support fibroblast proliferation and migration was also tested by seeding an 8 mm diameter, 1 mm thick scaffold made from a solution of 12% (w/v) SF and 0.2% (w/v) HAM, with a 5 µL drop of primary fibroblasts placed into the center of the scaffold top. Scaffolds were collected 2 and 7 days after seeding and immuno-stained with anti-vimentin to visualize fibroblasts (Figure 5, Supplementary Figure S8). On day 2, only a small number of fibroblasts have begun to migrate away from the initial seeding area. However, by day 7, nearly the entire top and bottom of the scaffold is fully covered in fibroblasts.

#### 3.2.3 Device Adherence

**FIGURE 6:**
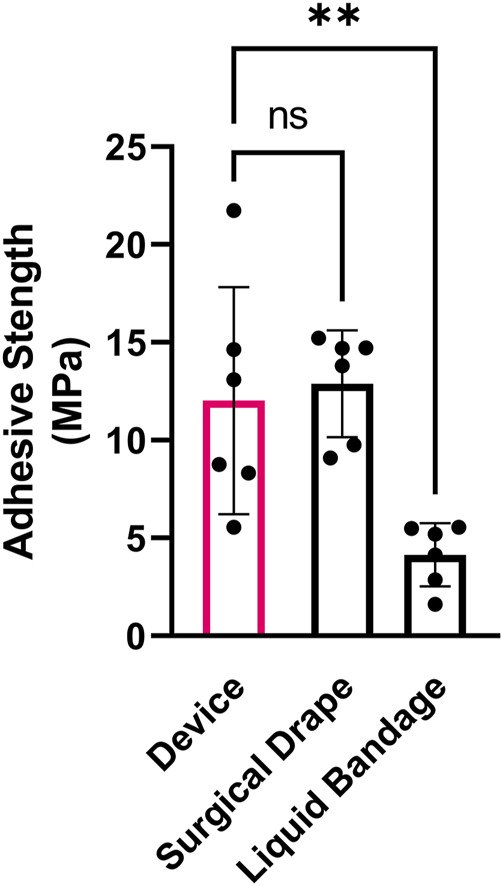
Device adhesive strength. Adhesive tensile strength of SF adhesive-coated complete device compared to a commercial surgical isolation drape and liquid bandage bonded to chamois leather (as skin analog) and pulled apart perpendicularly. n = 6; Mean ± SD; one-way ANOVA with Dunnett’s correction for multiple comparisons; **p < 0.01.

To test the adhesive strength of the device, 8 mm diameter complete devices, which included the tissue adhesive SF coated MNs, were adhered to chamois leather (as a skin analog) and pulled apart on a tensile testing fixture. The device demonstrated bonding of complete device to the skin analog in the MPa range, which was nearly identical to the adhesive strength of a commercially available adhesive surgical drape and significantly stronger than an over-the-counter liquid bandage formulation (Figure 6).

#### 3.2.4 Drug delivery capabilities

**FIGURE 7:**
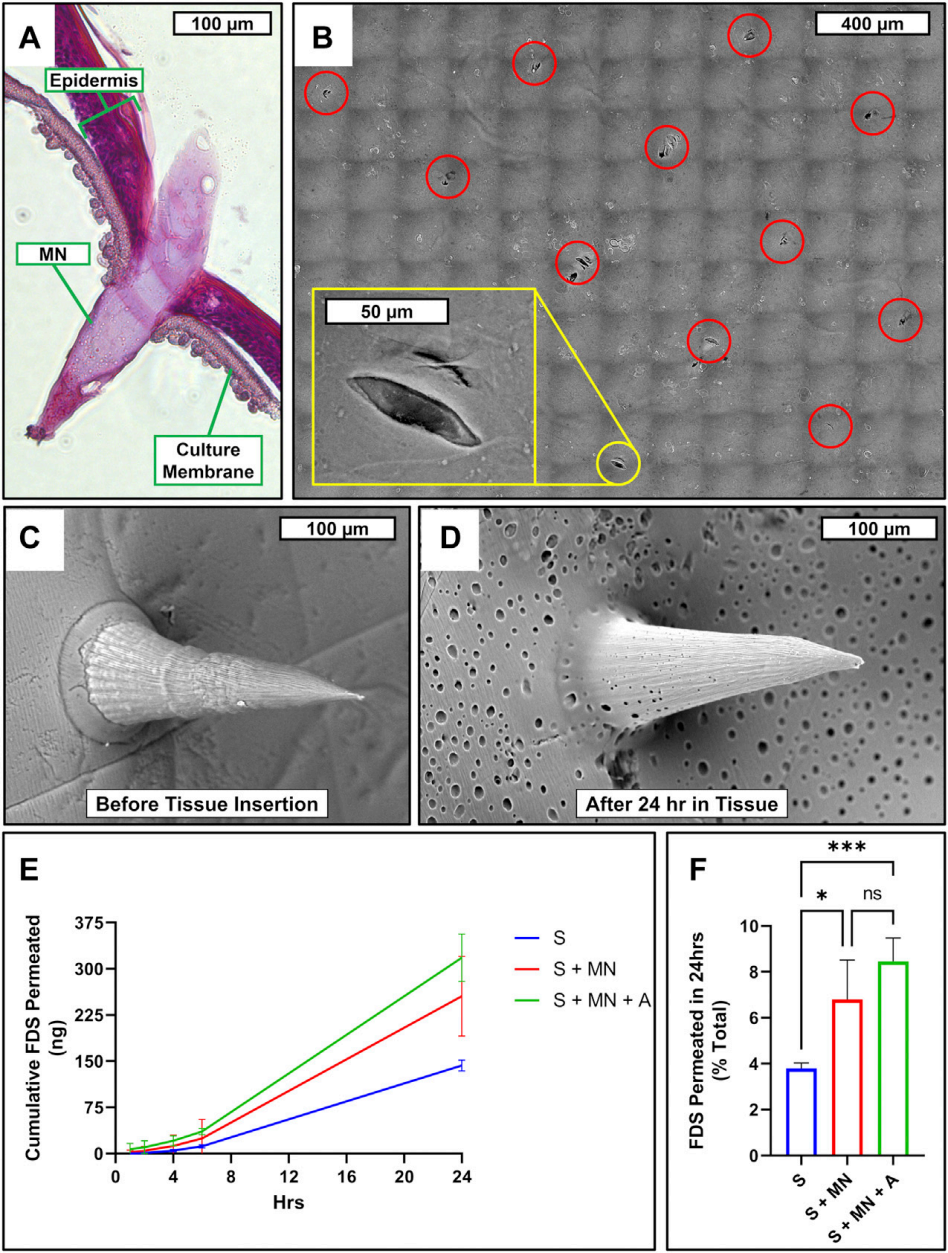
Microneedle skin penetration, drug permeability, and porosity. **(A)** Cross-sectional micrograph of RHE penetrated by SF/PVA MNs. **(B)** SEM micrograph mosaic of RHE surface following MN insertion and removal. Red circles highlight perforations caused by microneedle array; yellow inlay zooms in on a single perforation. SEM micrograph of SF/PVA MN film surface before **(C)** and after **(D)** being inserted in RHE for 24 h. **(E)** Cumulative permeated FDS at each timepoint and **(F)** percent of total applied FDS permeated over 24 h after application to the surface of each sample consisting of scaffold only (S; blue), scaffold with MN (S + MN; red), or scaffold with MN and SF adhesive coating (S + MN + A; green), which had been applied to the surface of an RHE. n = 4; one-way ANOVA with Tukey’s correction for multiple comparisons; *p < 0.05; ***p < 0.001. *FDS, fluorescein disodium; NS, not significant.*

To assess the skin penetration performance and drug permeability of the devices, 4 mm diameter complete devices were applied onto the surface of *in vitro* human epidermis models with gentle pressure from a sterile cotton swab. The MNs were found to easily penetrate the epidermis in a pattern consistent with the designed MN array (Figures 7A,B). After 24 h *in situ* residence in the human epidermis models, the water-soluble PVA in the MN array film surface appears to have dissolved away from the SF structure, as evidenced by the appearance of abundant pores (Figures 7C,D). To test the topical drug delivery effectiveness of the complete devices, fluorescein disodium was applied onto scaffolds containing scaffold only (S), scaffold with MNs (S + MN), and scaffold with MNs and the SF adhesive coating (complete device, S + MN + A). Relative to S alone, S + MN and S + MN + A both significantly increased topical drug permeation (Figures 7E,F).

### 3.3 Wound healing

**FIGURE 8:**
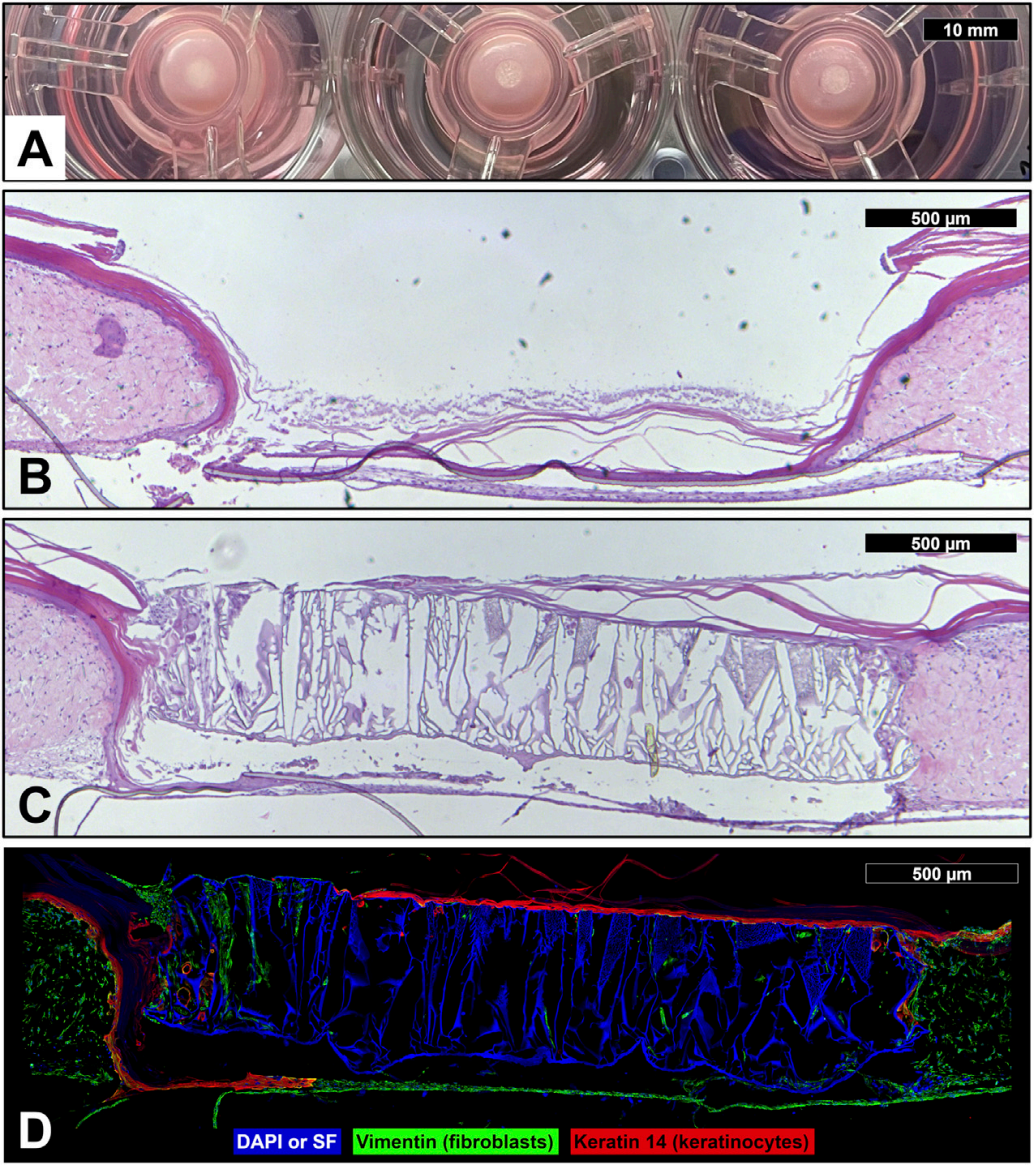
Device wound healing in vitro skin model. **(A)** Photograph of wounded human skin equivalents (3 mm full-thickness wound) treated with reconstruction devices at the start of the wound healing experiment (day 0). Micrographs of sectioned and hematoxylin and eosin stained full-thickness human skin equivalent which was wounded with a 3 mm biopsy punch, then left untreated as a control **(B)** or treated with a skin reconstruction device **(C)**, and allowed to heal for 20 days. **(D)** Immunofluorescent micrograph of wounded human skin equivalent treated with skin reconstruction device. DAPI and auto-fluorescent SF (blue); vimentin staining identifies fibroblasts (green); keratin 14 staining identifies keratinocytes (red).

To investigate the device’s effect on wound healing, *in vitro* full-thickness human skin equivalents were wounded with a 3 mm biopsy punch and a 3 mm diameter complete device was placed into the wound (Figure 8A), which was then allowed to heal for 20 days. The control tissue which was not treated with a device showed migration of epidermal keratinocytes across the base of the cell culture insert, but there remained an obvious tissue gap in the dermis which could not heal naturally (Figure 8B). The tissues treated with the devices provided a dermal scaffold and appeared to integrate well into the healing wound (Figure 8C). Epidermal keratinocytes (red) are seen forming a continuous epidermis across a majority of the device surface (blue) and fibroblasts (green) are observed migrating deep into the scaffold of the device (Figure 8D).

## 4 Discussion

With the deliberate intent to design a skin regeneration device for austere, resource-limited settings, each component was carefully considered and selected for incorporation into the final formulation. SF, a well-characterized, natural polymeric protein which is widely employed in biomedical applications, was chosen as the primary structural component for both the scaffold and MN array due to its biocompatibility, biodegradability, environmental stability, and the ease of tailoring its mechanical properties via structural changes (Vepari and Kaplan, 2007; Holland et al., 2019). PVA, a biocompatible, water-soluble synthetic polymer was added to the MN film to increase device porosity while transiently maintaining the initial mechanical robustness of the MN. Specifically, the microneedles are non-porous at the time of deployment but reveal micropores within 24 h as the PVA microdroplets dissolve out of the MN array. Although not tested in our studies, previous reports suggest that the addition of PVA to SF may also improve cytocompatibility relative to SF alone (Ulloa Rojas et al., 2022).

The final component of the complete device, HAM, is a hyaluronan-conjugate previously developed by our group and shown to decrease oxidative stress (Arrigali and Serban, 2022). HAM builds on HA’s excellent intrinsic biocompatibility and ease of chemical modification (Serban and Skardal, 2019; Graça et al., 2020; Marinho et al., 2021). HA, a natural polymeric glycosaminoglycan, has been extensively characterized for its role in wound healing (Chen and Abatangelo, 1999; Schanté et al., 2011; Neuman et al., 2015). Endogenous HA is known to have size-dependent biological effects on wound healing processes, with high molecular weight HA having been reported to be anti-inflammatory, anti-angiogenic, and anti-proliferative, while low molecular weight HA has been found to be pro-inflammatory, pro-angiogenic, and pro-wound healing (Chen and Abatangelo, 1999; Aya and Stern, 2014; Neuman et al., 2015). Interestingly, each of these extremes possess both desirable and undesirable properties in the context of skin reconstruction. While the exact range of what is considered high- and low-molecular weight HA varies across studies, our HAM characterization data indicates that the conjugate’s molecular weight (192 KDa) appears to fall consistently between the two groups, which may reduce the risk of significant detrimental effects seen at each extreme, and in our experiments showed beneficial biological effects. However, if pertinent, adaptation of HAM synthesis to generate significantly higher or lower molecular weight forms could be explored in future iterations to target the various biological effects as needed.

The overall assembly of the skin reconstruction device follows a deconstructed and easily scalable method which separately produces a porous SF/HAM scaffold adhered to a MN array made from a SF/PVA blend. The assembled device is rendered water insoluble via a simple physical cross-linking process, dried, then a thin coating of adhesive SF (Johnston et al., 2018) is sprayed onto the surface of the MNs and allowed to dry. Upon MN insertion into tissue, the adhesive is rehydrated by physiological moisture, forming a strong bond between the device and tissue surface. In contrast to traditional grafts and skin reconstruction systems, the self-adherent nature of this device eliminates the need for sutures or traditional invasive fixation methods and can be deployed without the need for trained medical personnel. Our comparison with a commercially available adhesive surgical drape and an over-the-counter liquid bandage formulation offers some insight into the adhesiveness of our device and suggests it should provide adequate adhesion *in situ*. Although this study did not investigate shorter adhesion times, our previously published work (Johnston et al., 2018) investigated the properties of this SF adhesive given shorter setting times. This work assessed both wet adhesive (60 s equilibration) and partially dried adhesive (3 h equilibration) strength, which could be used to extrapolate some level of confidence in the time-dependent adhesive potential of the device.

Our mechanical characterization data indicate that a device formulation comprising of 12% (w/v) SF and 0.2% (w/v) HAM provides the ideal balance between mechanical strength and ROS protection. It is important to underline that our device design was focused on the development of a robust, easy-to-handle system that would provide a sufficient mechanical barrier during handling and wound healing, while facilitating tissue-mediated device degradation and resorption, and a match of the exact mechanical properties of native skin was not deemed relevant. As is, the device provides a sufficiently strong protective barrier which allows for reasonable flexibility and ease of motion of wounded tissue throughout the device handling and healing process. Although we did not investigate this in our study, the device composition leads us to expect device degradation as the wound heals and subsequent studies will be focused on assessing the exact timeline of this process.

Based on the well-understood properties and previous studies of the constituent materials, our devices are expected to be fully biocompatible, although this will also need to be confirmed in subsequent preclinical studies. The data presented herein supports our expectations as standardized skin irritation tests following OECD TG439 guidelines showed that exposure to neither the device nor excess HAM resulted in any decrease in epidermal tissue viability relative to control. This *in vitro* organotypic test is considered equivalent to traditional *in vivo* preclinical models and has been shown to be accurately predictive of *in vivo* performance (OECD, 2021).

The antioxidant effects of HAM, and its ability to improve Dmet cellular internalization over unconjugated Dmet, have been previously described by our group in cochlear cells (Arrigali and Serban, 2022). Although Dmet is intrinsically antioxidant, it is not readily internalized by cells in efficacious concentrations. By conjugating Dmet to HA, we can leverage HA-receptor mediated internalization to increase intracellular Dmet. This effect is clearly demonstrated in this study, which shows ROS protecting effects by HAM but not by equivalent concentrations of unconjugated Dmet. As literature reported *in vivo* data indicates that elevated and sustained ROS levels are detrimental to wound healing (Dunnill et al., 2017), we evaluated the effect of HAM on primary human fibroblasts treated with menadione, a compound which strongly induces cellular production of ROS (Singh and Husain, 2018), then quantified cellular metabolic activity by MTS reduction assay, which is commonly used as a cell viability assay. The results indicate that HAM strongly protects against cellular damage by ROS, while equivalent amounts of CMHA, Dmet, or a blend of the two in the unconjugated form, do not.

Next, since fibroblast migration and proliferation are critical steps in proper wound healing (Saghazadeh et al., 2018), we sought to evaluate these processes in our devices. Our data show that the SF/HAM scaffold facilitates generous fibroblast migration and proliferation *in vitro*. We also show that the skin reconstruction device is able to integrate into *in vitro* human skin equivalents following full-thickness (epidermis and dermis) wounding. Due to the thinness of the *in vitro* human skin equivalent used, a device made from 0.5 mm thick scaffold was used in place of the 1.0 mm thick scaffold used in the rest of the study. This was needed to allow the adjacent epidermal keratinocytes to migrate over the device in a manner more representative of the real-world conditions expected in deeper *in vivo* wounds. This preliminary data showed that, despite the lack of underlying tissue in this *in vitro* model (relative to *in vivo* skin), within 20 days the device was able to integrate into the tissue to act as a dermal scaffold within the wound. Epidermal keratinocytes migrated across the surface and continued to produce a protective stratum corneum, while fibroblasts had begun to migrate deep into the scaffold. We anticipate that, in an extended form of this study, the wound healing response will overlap with proteolytic degradation (Holland et al., 2019) of the device. This process should involve the device being increasingly replaced by the endogenous extracellular matrix components, such as collagen and elastin which are secreted by the native fibroblasts, and we hope to investigate these device integration aspects in subsequent studies. Within austere environments, existing skin substitutes, if available, would likely only be applied days after the initial wounding once the patient reaches a properly equipped medical facility. Given that the device detailed in this study is intended to offer a rapid, more immediate treatment option than currently exists in austere environments, we opted to compare this device’s *in vitro* wound healing performance relative to a control wound which received no treatment as opposed to an existing skin reconstruction product. We postulate this comparison to be more representative of the actual outcomes which would be seen in the field.

Another essential consideration for the device design was its ability to enable topical drug delivery. This feature is intended to allow patient/situationally-specific antibiotics, local anesthetics, or any other therapeutics deemed appropriate for care, to be simply applied to the surface of the device to directly treat the targeted tissue, thus reducing or eliminating the adverse effects associated with systemic treatments (Holtman and Jellish, 2012; Carmichael et al., 2018; Negut et al., 2018). Previous studies have also suggested that MNs, in addition to enhancing drug permeation, may also inherently aid wound healing by stabilizing the wound or mechanically promoting debridement and cell proliferation (Barnum et al., 2020). Our approach to facilitating topical drug treatments was to incorporate a dense MN array as well as macro- and micro-pores into the device design. The results of this study show that our custom MN arrays can fully and efficiently penetrate the tough epidermis and even break though the underlying polycarbonate membrane of the cell culture inserts used in the *in vitro* epidermis model. Although this study utilized *in vitro* epidermis models commonly used for preliminary drug permeation and skin barrier assessments (Agonia et al., 2022) and SF MN have been previously shown to be effective in animal models (Tsioris et al., 2012; Stinson et al., 2017), future *in vivo* or skin explant studies are required to confirm efficacy of this device’s MN array. We also show that the MN array with adhesive coating more than doubles drug delivery across the epidermis relative to the scaffold alone. Although only about 8% of the total drug applied permeated over the initial 24-h period, we suspect that a significant portion of the drug was absorbed into the scaffold and, upon saturation, subsequent doses applied to the device would likely result in significantly more drug permeating more rapidly. The results also indicate that within 24 h of being inserted into a tissue, the PVA component of the MN film dissipates leaving the insoluble SF film with sufficient porosity to facilitate drug permeation and cellular infiltration.

Of note, in line with the Food and Drug Administration’s initiative to reduce animal testing and promote the use of qualified alternative methods for product testing (FDA, 2023), this study employed validated, standardized *in vitro* testing methods for the initial assessments of cytocompatibility, tissue interaction, and wound healing efficiency. Our data now positions these devices to be confidently advanced to subsequent targeted preclinical studies that would seek to address their biodegradation, bioresorption, and overall *in vivo* performance.

## 5 Conclusion

Altogether, this study describes the conceptualization, development, and proof of concept of a unique, user-friendly, effective, biomaterial-based skin reconstruction system specifically targeting the needs of patients in resource-limited settings. We demonstrated that this device is cytocompatible and non-irritant, displays favorable mechanical properties, protects cells from ROS damage, facilitates cell migration and wound healing *in vitro*, and improves topical drug permeability. The device formulation is expected to translate into a biocompatible, highly stable, cold-chain independent product, although these aspects will be specifically addressed in subsequent studies. Future iterations of the device concept presented herein will seek to address the issue of eschar and wound debridement via similar minimalist approaches. However, in its current form, the device appears to represent a viable option for secondary care centers and rural clinics. Overall, this work highlights the unique considerations associated with the generation of treatment options for austere environments where minimalist but versatile designs may be better suited than traditionally innovated concepts.

## Data Availability

The raw data supporting the conclusions of this article will be made available by the authors, without undue reservation.
