# Supplementary material for: A versatile, bioengineered skin reconstruction device designed for use in austere environments
Source: Front Bioeng Biotechnol. 2023 Jun 8;11:1208322. doi: 10.3389/fbioe.2023.1208322 (PMC10285514; doi:10.3389/fbioe.2023.1208322)
Supplement: Supplementary file 1 [file DataSheet1.PDF]

Supplementary Materials

A Versatile, Bioengineered Skin Reconstruction Device Designed for Use in Austere Environments

Joachim G. S. Veit, Morgan Weidow, Monica A. Serban*

***Correspondence:** Monica A. Serban: monica.serban@umontana.edu

1. SUPPLEMENTARY MATERIALS AND METHODS

1.1. HAM Molecular Weight Determination

Molecular weight and polydispersity of HA, CMHA, and HAM were determined using multi-angle light scattering (MALS). Refractive index (RI) increment (dn/dc) for each was determined using batch dn/dc determinization on an Optilab RI detector with ASTRA software (Wyatt, Santa Barbara, CA) as instructed by the manufacturer (Wyatt Technology Corporation, 2016). Briefly, five known concentrations (between 0.1 - 1.0 mg mL^{-1}) of each HA polymer were prepared in the mobile phase subsequently used for HPLC and sequentially injected directly into the flow cell of the RI detector with a syringe pump. The entire process from weighing and reconstituting lyophilized sample to dilution and batch dn/dc determination was performed in triplicate for each sample and the average dn/dc reported by the ASTRA software was used as the input parameter for molecular weight determination by MALS.

Size exclusion chromatography (SEC)-MALS was then performed on each HA polymer using a miniDAWN (MALS) and Optilab (RI) detector combination (Wyatt Technology) which were connected to the output of an Agilent 1260 Infinity II HPLC system. The mobile phase was HPLC-grade water with 0.02% sodium azide (vacuum filtered at 0.1µm prior to use) flowing at 1.0 mL min^{-1} though a 6.0 - 10,000 kDa 8 µm PL Aquagel-OH Mixed-H 7.5 x 300 mm SEC column (Agilent, PL1149-6800). 50 µL of 0.2 mg mL^{-1} polymer solution was injected. SEC-MALS was performed in triplicate for each sample and the molecular weights and polydispersity were reported by ASTRA software.

1.1. Dmet Conjugation Efficiency

HAM (10 mg mL^{-1}) was hydrolyzed by combining sample (100 µL) with HCl (100 µL, 12 M) in a glass vial which was nitrogen flushed before sealing and autoclaved for two sequential 40 min liquid cycles (121 °C, 1.2 bar). This was then neutralized with NaOH (200 µL, 6 M) and pH 7.0 potassium phosphate buffer (100 µL, 0.1 M), before filtering at 0.22 µm.

The samples then underwent o-phthalaldehyde (OPA) derivation and HPLC detection using a previously described method (Choung et al., 2013) that had been adapted for our purpose. A derivation reagent was made by combining methanol (0.75 mL), boric acid (14.25 mL, 0.4 M) at pH 10.4 using potassium hydroxide (8 M), mercaptoacetic acid (0.3 mL), and OPA (150 mg). This was brought to pH 10.4 with potassium hydroxide (8 M) and filtered at 0.22 µm. Hydrolyzed sample (100 µL) was combined with isopropanol (110 µL) and derivation reagent (40 µL). This was then incubated at 60 °C for 15 min before transferring to an HPLC vial for analysis.

Reverse-phase HPLC was performed using a 100 x 4.6 mm Gemini 3µm C18 110Å column (Phenomenex, 00D-4439-E0) protected by a SecurityGuard C18 4 x 3.0mm cartridge (Phenomenex, AJ0-7597) at 30 °C on an Agilent 1260 Infinity II HPLC system. Sample (10 µL) was injected at a flow rate of 1.0 mL min^{-1}. The mobile phase composition and gradient is detailed in **Supplementary Table S1**. The OPA-Dmet product was detected at 10.9 min by UV-Vis detection at 330 nm and the concentration was determined by area under the curve against a Dmet standard.

The entire determination process from weighing and reconstituting lyophilized sample and standards to hydrolysis, derivation and HPLC was performed in triplicate.

2. SUPPLEMENTARY RESULTS

2.1. Characterization of HAM

Our novel polymer, HAM, is the component primarily responsible for antioxidant and wound healing properties of the device. To supplement our previous work and better understand this component, we sought to describe several of its physical and biological properties.

2.1.1. Physical Properties

SEC-MALS was employed to determine the polymer properties for each step of the synthesis including the HA starting material, the carboxymethylated HA (CMHA) intermediate, and the final HA-methionine-conjugate (HAM) product (**Supplementary Figure S9**). The HA starting material was determined to have a monodisperse (Đ = 1.079) weight average molecular weight (M_w) of 678.8 kDa (**Supplementary Figure S4**, **Supplementary Table S2**). The first synthesis targets the enrichment of HA with additional carboxymethyl moieties to provide additional reaction sites for conjugation of Dmet in subsequent steps. Quantitative ^{1}H-NMR (**Supplementary Figure S1**) was used to determine a carboxymethylation efficiency of 51.3 ± 0.58% (mean ± SD) of the available sites (one possible site per HA disaccharide unit). This reaction led to a more polydisperse polymer (Đ = 1.402) and a large decrease in M_w (220.8 kDa) (**Supplementary Figure S4**, **Supplementary Table S2**). The next synthesis step conjugated Dmet to CMHA to form HAM. This resulted in a much smaller decrease in M_w (192.9 kDa) relative to CMHA and similar polydispersity (Đ = 1.325). Successful Dmet conjugation was confirmed with ^{1}H-NMR as previously described (Arrigali and Serban, 2022). Conjugation efficiency of Dmet to CMHA was found to be 9.76 ± 0.054% (w/w) of the total mass of HAM (**Supplementary Table S2**).

2.1.2. *HAM Cytocompatibility*

The cytocompatibility of HAM was determined by skin irritation testing following OECD TG439 guidelines. HAM in solution (10 mg mL^{-1}) was applied to in vitro 3D human epidermis models. HAM showed no evidence of loss of tissue viability relative to the untreated control and remained robustly above the guidelines threshold of 50% which classifies HAM as a non-irritant (**Supplementary Figure S5A**).

3. SUPPLEMENTARY FIGURES

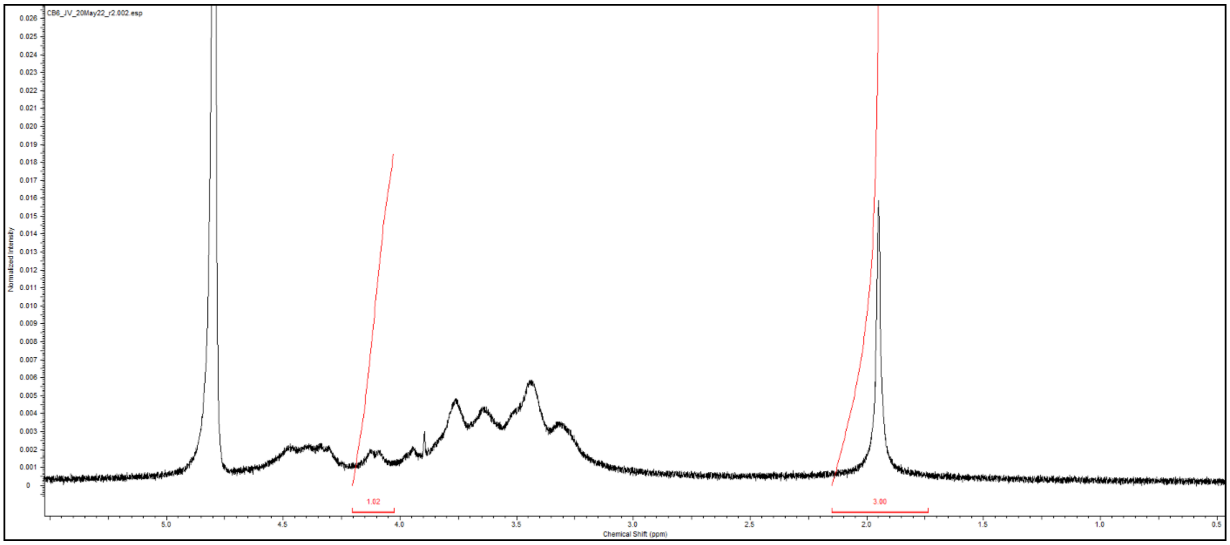

Supplementary Figure S1. Proton NMR of CMHA used to quantify efficiency of carboxymethylation of HA. Performed in triplicate, methylene protons from the carboxymethyl group (@ 4.1 ppm) were normalized to the methyl protons of the N-acetylglucosamine (@ 1.95 ppm) to determine the efficiency of carboxymethylation, which was found to be 51.3 ± 0.58% (mean ± SD) of the available sites (one site per HA disaccharide unit). *CMHA, carboxymethyl hyaluronic acid.*

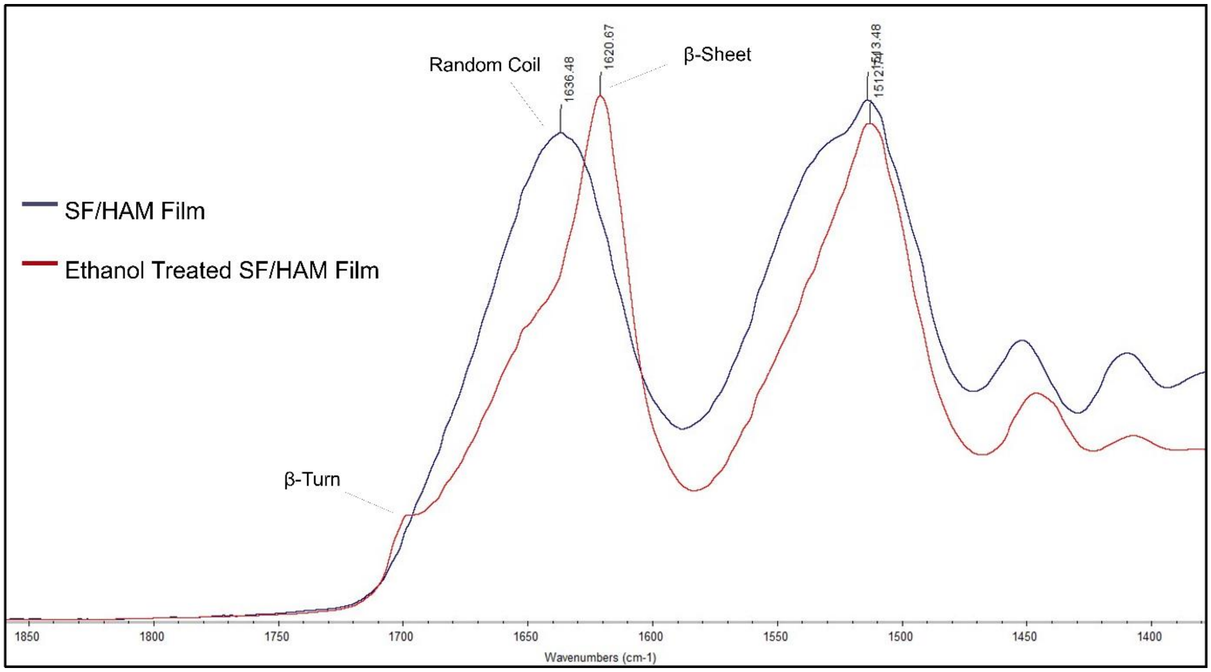

Supplementary Figure S2. FTIR spectra of a film cast from 12% SF with 0.2% HAM before (blue) and after (red) treatment with 90% ethanol to induce β-sheet and β-turn formation in SF. Absorption axis (y axis) left unlabeled since the two samples were arbitrarily scaled to equal height for figure clarity rather than sharing a common scale. *HAM, hyaluronic acid D-methionine; SF, silk fibroin.*

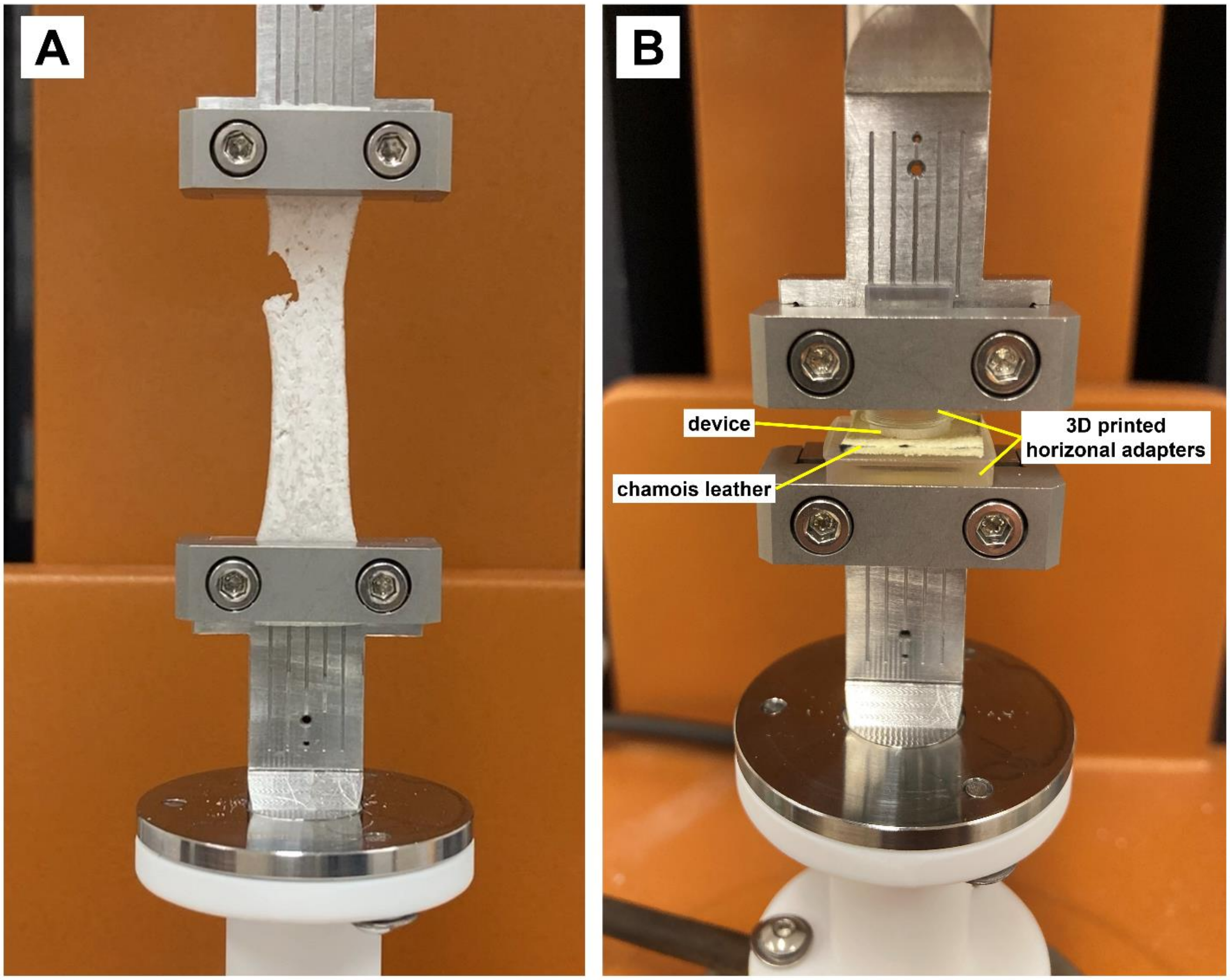

Supplementary Figure S3. (**A**) Photograph of tension fixture containing a SF/HAM scaffold mid-tension testing. (**B**) Photograph of setup for device adhesive testing. The SF adhesive coated MNs on the device are adhered to the chamois leather. The chamois leather and top of the device are adhered to the adapters with cyanoacrylate glue. The device and chamois leather are pulled apart until the glue fails. *HAM, hyaluronic acid D-methionine; SF, silk fibroin.*

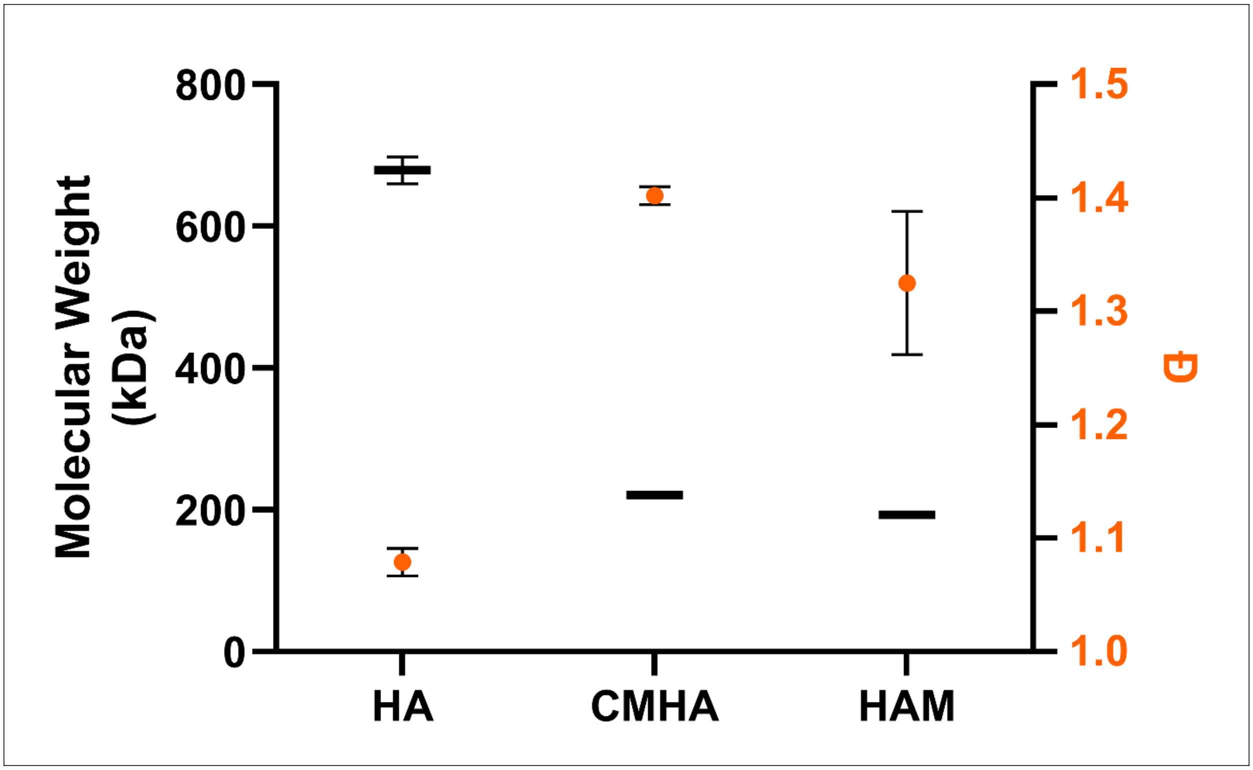

Supplementary Figure S4. Molecular weight of HA, CMHA, and HAM. Weight average molecular weight (black line, left y-axis) and dispersity index (Đ, orange circle, right y-axis) of HA starting material, CMHA intermediate, and HAM final product. Lines show mean ± SD; n = 3. *CMHA, carboxymethyl HA; Đ, dispersity index; HA; hyaluronic acid; HAM, HA D-methionine.*

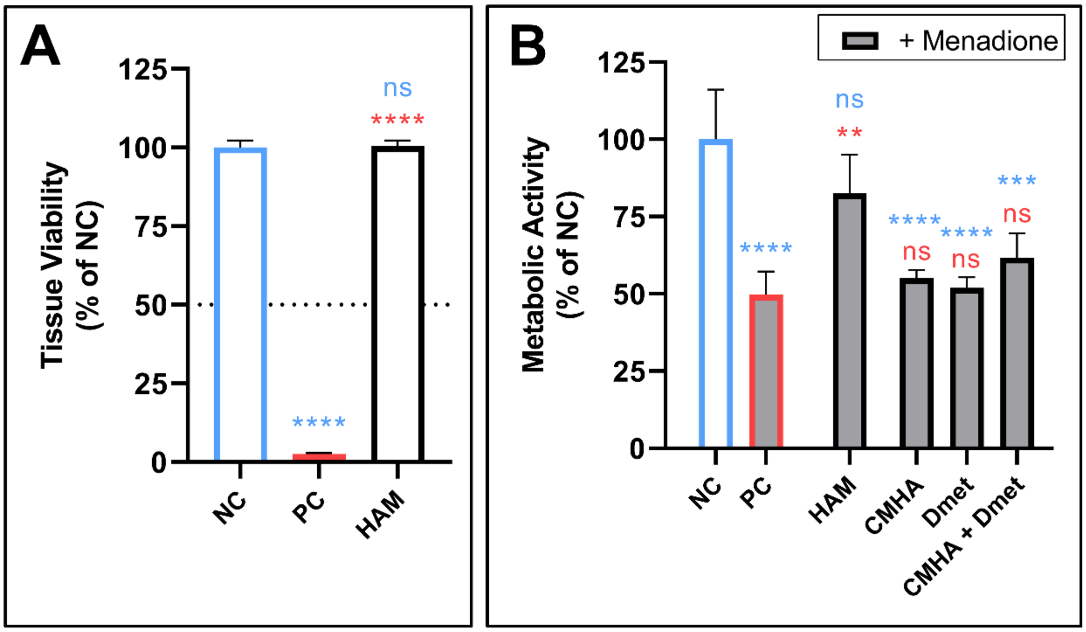

Supplementary Figure S5. (**A**) Skin irritation test (following OECD TG439) of NC (vehicle), PC (5% SDS), and HAM (10 mg mL^{-1}) applied to the surface of 3D in vitro human epidermis. Dotted line indicates cutoff threshold indicating whether test compound is considered a possible irritant. n = 3; Mean + SD; one-way ANOVA with Tukey's correction for multiple comparisons vs NC (blue) or PC (red); ****p < 0.0001. ROS protection by (**B**) 1 mg mL^{-1} HAM. MTS assay measuring metabolic activity of primary human fibroblasts treated for 24 hrs with HAM, or equivalent concentrations of CMHA alone, Dmet alone, or a blend of CMHA + Dmet. Followed by 5 hr treatment with 10 µM menadione to generate ROS (indicated by grey fill). n = 4; Mean + SD; one-way ANOVA with Tukey's correction for multiple comparisons vs NC (blue) or PC (red); **p < 0.01, ***p < 0.001, ****p < 0.0001. *CMHA, carboxymethyl HA, Dmet, D-methionine; HA, hyaluronic acid; HAM, HA Dmet conjugate; MTS, (3-(4,5-dimethylthiazol-2-yl)-5-(3-carboxymethoxyphenyl)-2-(4-sulfophenyl)-2H-tetrazolium); NC, negative control; NS, not significant; PC, positive control; ROS, reactive oxygen species.*

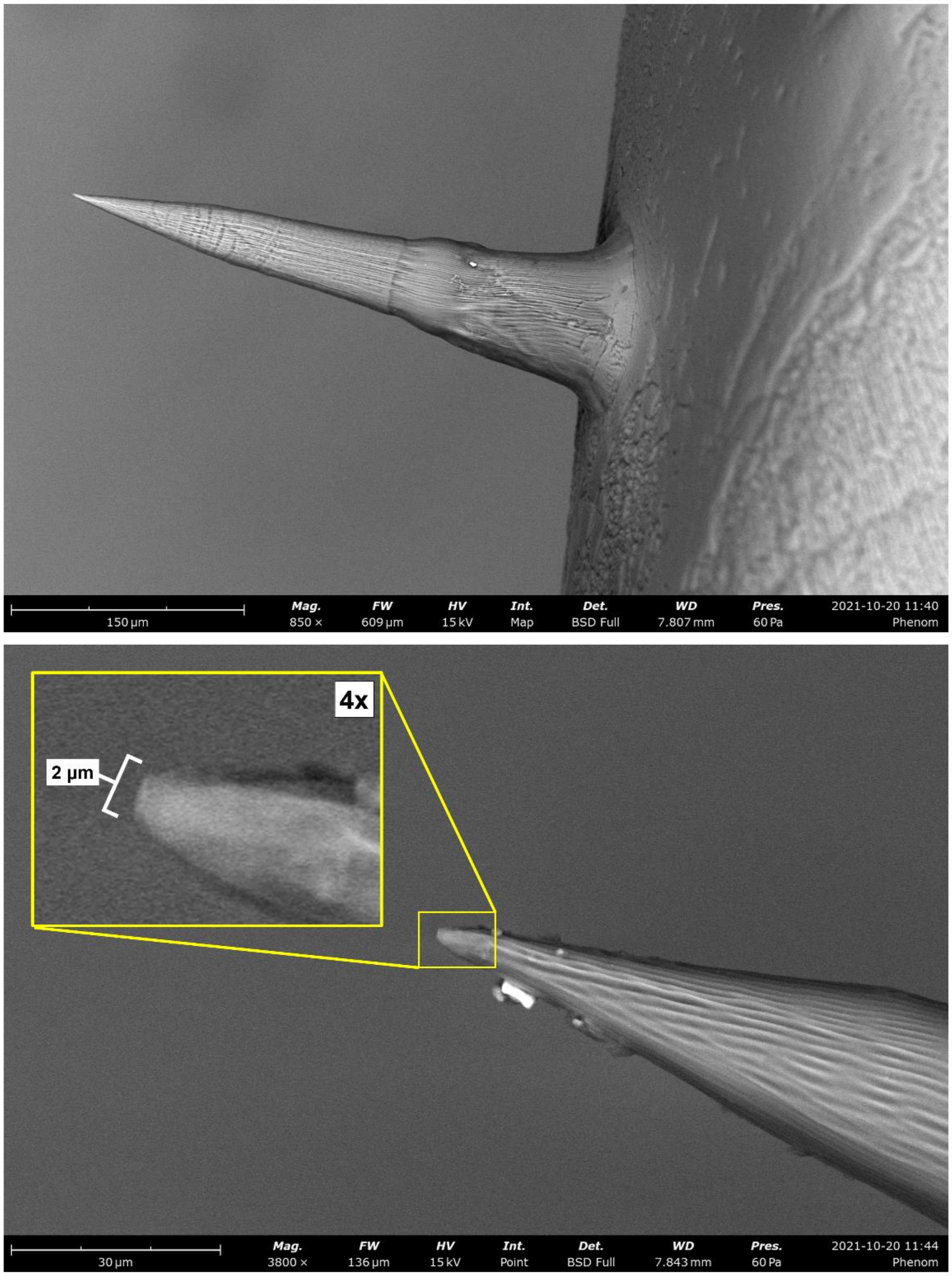

Supplementary Figure S6. SEM micrograph of SF/PVA microneedles showing typical tip diameter. *PVA, poly-vinyl alcohol; SF, silk fibroin.*

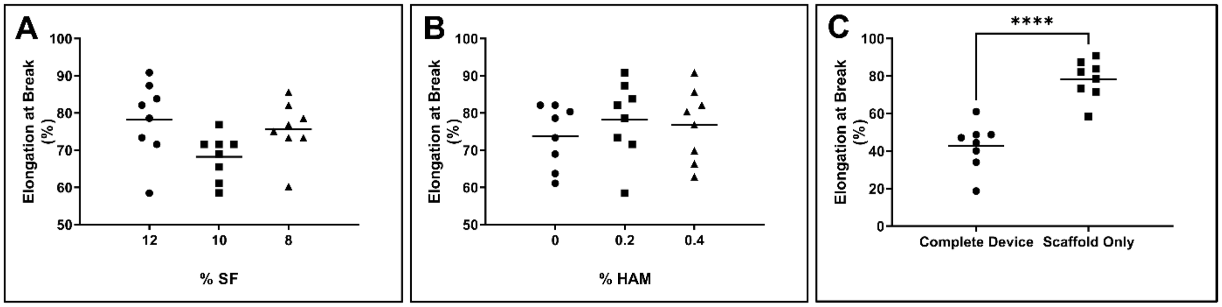

Supplementary Figure S7. Elongation at break for scaffolds made with varying SF concentrations with 0.2 % HAM (**A**), scaffolds made with 12 % SF and varying HAM concentrations (**B**), and complete device compared to scaffold only (**C**). Neither SF concentration or HAM content made any significant difference in elongation at break. The complete device had a shorter elongation at break, likely due to the lower elasticity of the microneedle film. (**A**-**B**) n = 8; One-way ANOVA with Tukey's correction for multiple comparisons. (**C**) n = 8; Student's t-test; ****$p < 0.0001$. *HAM, hyaluronic acid D-methionine; SF, silk fibroin.*

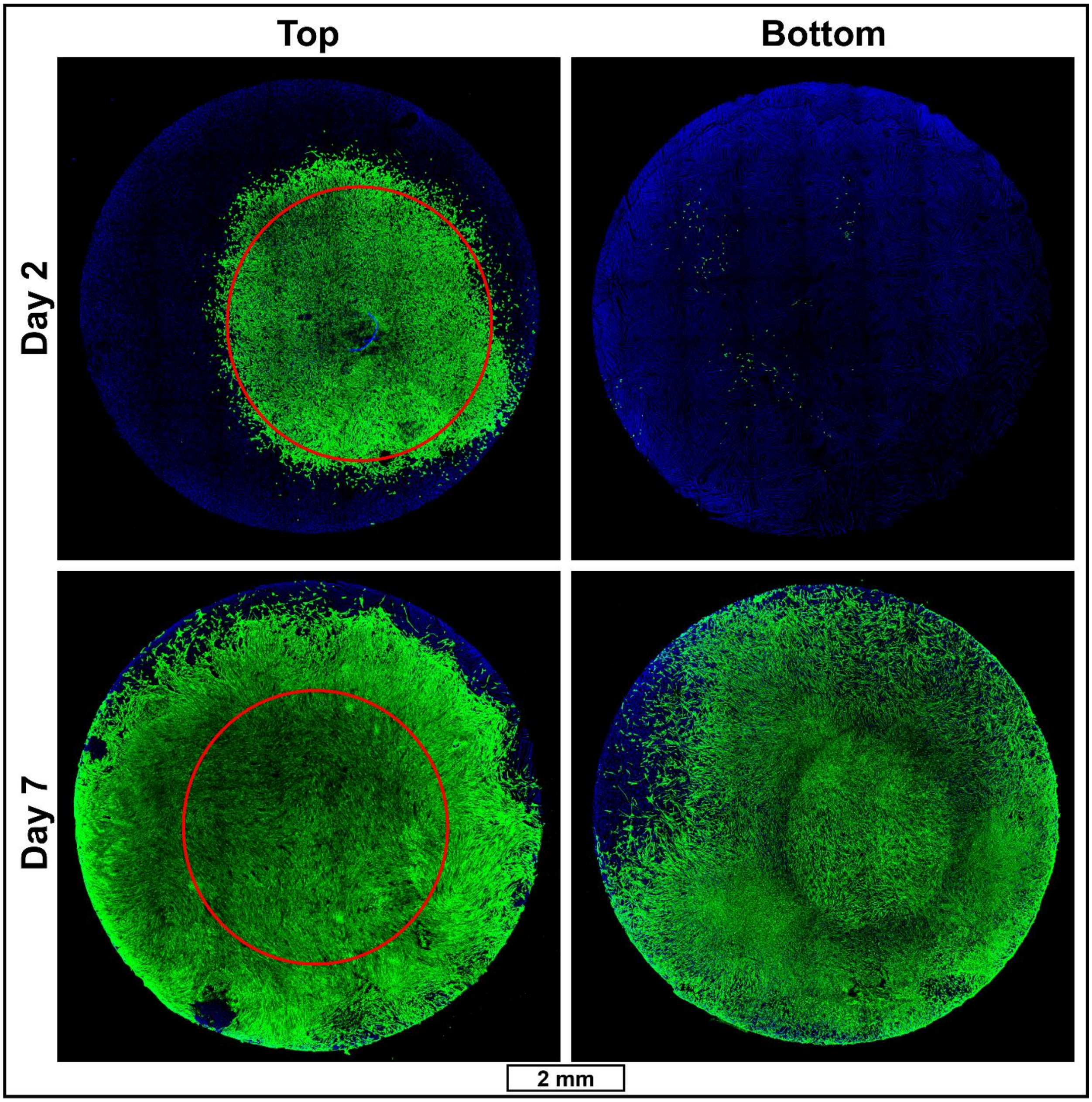

Supplementary Figure S8. Replicate data for fibroblast migration in SF/HAM scaffold. Additional immunofluorescent confocal micrographs of 8 mm diameter (1 mm thick) SF/HAM scaffolds cultured for 2 (top row) or 7 (bottom row) days following initial seeding with a 5 µL drop of primary fibroblasts directly onto the top center surface of the scaffold. Red circle shows approximate initial seeding area to illustrate extent of migration. Scale bar applies to all images. DAPI (nuclear stain) and auto-fluorescent SF (blue); vimentin staining identifies fibroblasts (green). *HAM, hyaluronic acid D-methionine; SF, silk fibroin.*

Supplementary Table S1. Mobile phase gradient for HPLC determination of D-methionine content.

**Eluent A**	70:25:5 methanol:water:acetic acid + 5 g L^{-1} sodium 1-heptanesulfonate
**Eluent B**	water

Step #	Time (min)	% Eluent A	% Eluent B
1	0	50	50
2	4	50	50
3	5	75	25
4	6.5	75	25
5	10.5	100	0
6	12.5	100	0
7	13	50	50
8	16	50	50

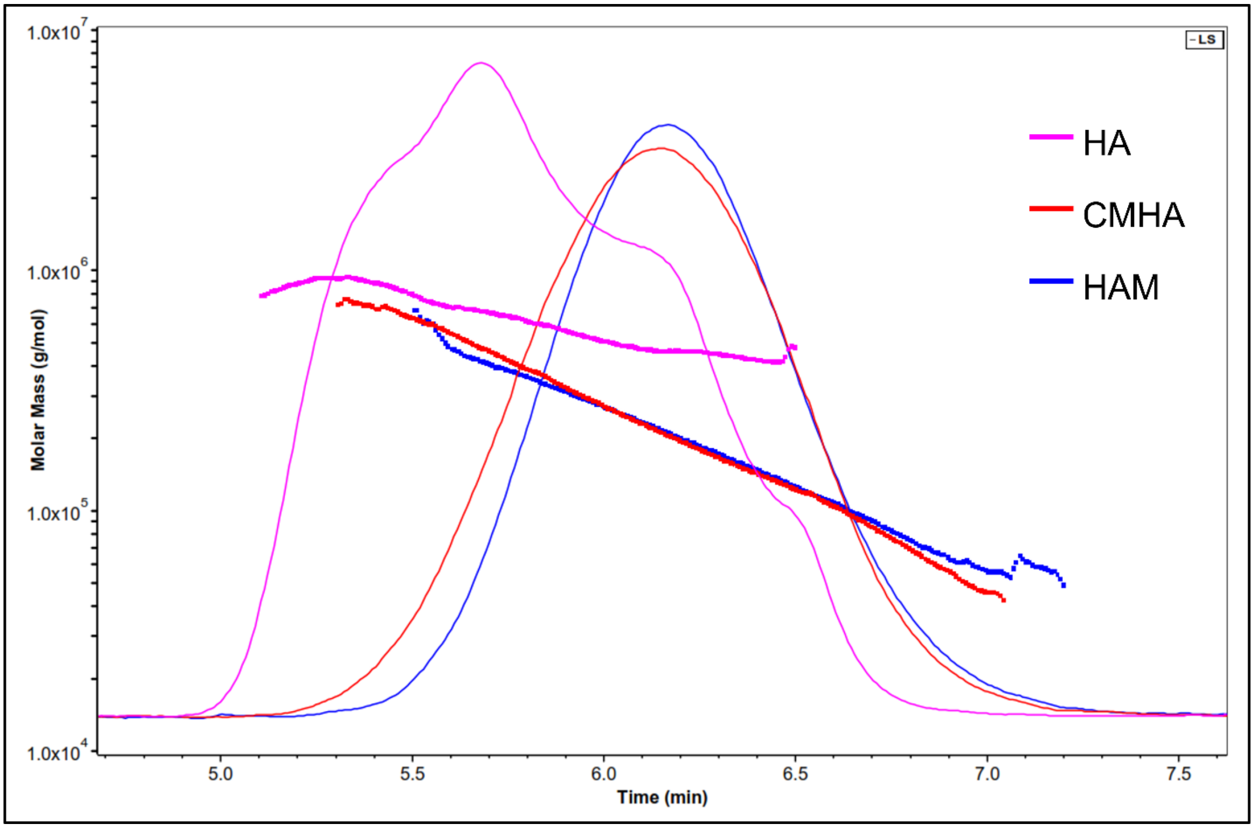

Supplementary Figure S9. MALS plots of HA and derivatives. Representative SEC-MALS plots of HA, CMHA (intermediate), and HAM. CMHA, carboxymethyl HA; Dmet, D-methionine; HA, hyaluronic acid; HAM; HA Dmet, MALS, multi-angle light scattering SEC, size-exclusion chromatography.

Supplementary Table S2. Refractive index increment, molecular weight, and Dmet content in each step of the HAM synthesis.

	dn / dc	M_w	M_n	M_p	M_z	Đ	Dmet
	mL g^{-1}	kDa				M_w / M_n	% (w/w)
**HA**	0.1482 ± 0.0012	678.8 ± 19.1	629.3 ± 11.8	693.7 ± 2.1	731.3 ± 29	1.079 ± 0.012	-
**CMHA**	0.1522 ± 0.0035	220.8 ± 1.7	157.6 ± 1.9	173 ± 2.2	305.6 ± 4.4	1.402 ± 0.008	-
**HAM**	0.1656 ± 0.0034	192.9 ± 1.7	145.8 ± 5.8	181.5 ± 3.0	245.4 ± 12.4	1.325 ± 0.063	9.76 ± 0.054

n = 3; Mean ± SD. *CMHA, carboxymethyl HA; Đ, dispersity index; Dmet, D-methionine; dn / dc, refractive index increment; HA, hyaluronic acid; HAM, CMHA-Dmet conjugate;* M_w, *weight average molecular weight;* M_n, *number average molecular weight;* M_p, *concentration peak apex molecular weight;* M_z, *z average molecular weight (third moment).*

4. SUPPLEMENTARY REFERENCES

Arrigali, E. M., and Serban, M. A. (2022). Development and Characterization of a Topically Deliverable Prophylactic Against Oxidative Damage in Cochlear Cells. *Front. Pharmacol.* 13, 1–14. doi: 10.3389/fphar.2022.907516.

Choung, M. C., J., C., J.W., H., E., K., W., A., F., A. D., et al. (2013). High Performance Liquid Chromatopgraphy of Gentamicin Sulfate Reference Standards and Injection USP. *Int. J. Pharm. Anal.* 4, 25–29. Available at: http://www.bioinfopublication.org/jouarchive.php?opt=&jouid=BPJ0000243.

Wyatt Technology Corporation (2016). Batch dn/dc Measurements (TN4000). 1–8.
